# Differential effects of group III metabotropic glutamate receptors on spontaneous inhibitory synaptic currents in spine-innervating double bouquet and parvalbumin-expressing dendrite-targeting GABAergic interneurons in human neocortex

**DOI:** 10.1093/cercor/bhac195

**Published:** 2022-06-07

**Authors:** Istvan P Lukacs, Ruggiero Francavilla, Martin Field, Emily Hunter, Michael Howarth, Sawa Horie, Puneet Plaha, Richard Stacey, Laurent Livermore, Olaf Ansorge, Gabor Tamas, Peter Somogyi

**Affiliations:** Department of Pharmacology, University of Oxford, Oxford OX1 3QT, UK; Department of Pharmacology, University of Oxford, Oxford OX1 3QT, UK; Department of Pharmacology, University of Oxford, Oxford OX1 3QT, UK; Department of Pharmacology, University of Oxford, Oxford OX1 3QT, UK; Department of Pharmacology, University of Oxford, Oxford OX1 3QT, UK; Department of Pharmacology, University of Oxford, Oxford OX1 3QT, UK; Department of Neurosurgery, John Radcliffe Hospital, OUH NHS Foundation Trust, Oxford OX3 9DU, UK; Department of Neurosurgery, John Radcliffe Hospital, OUH NHS Foundation Trust, Oxford OX3 9DU, UK; Department of Neurosurgery, John Radcliffe Hospital, OUH NHS Foundation Trust, Oxford OX3 9DU, UK; Nuffield Department of Clinical Neurosciences, University of Oxford, Oxford OX3 9DU, UK; Department of Physiology, Anatomy and Neuroscience, University of Szeged, 6726 Szeged, Hungary; Department of Pharmacology, University of Oxford, Oxford OX1 3QT, UK

**Keywords:** cell type, GABA_A_ receptor, inhibition, L-AP4, presynaptic receptor

## Abstract

Diverse neocortical GABAergic neurons specialize in synaptic targeting and their effects are modulated by presynaptic metabotropic glutamate receptors (mGluRs) suppressing neurotransmitter release in rodents, but their effects in human neocortex are unknown. We tested whether activation of group III mGluRs by L-AP4 changes GABA_**A**_ receptor-mediated spontaneous inhibitory postsynaptic currents (sIPSCs) in 2 distinct dendritic spine-innervating GABAergic interneurons recorded in vitro in human neocortex. Calbindin-positive double bouquet cells (DBCs) had columnar “horsetail” axons descending through layers II–V innervating dendritic spines (48%) and shafts, but not somata of pyramidal and nonpyramidal neurons. Parvalbumin-expressing dendrite-targeting cell (PV-DTC) axons extended in all directions innervating dendritic spines (22%), shafts (65%), and somata (13%). As measured, 20% of GABAergic neuropil synapses innervate spines, hence DBCs, but not PV-DTCs, preferentially select spine targets. Group III mGluR activation paradoxically increased the frequency of sIPSCs in DBCs (to median 137% of baseline) but suppressed it in PV-DTCs (median 92%), leaving the amplitude unchanged. The facilitation of sIPSCs in DBCs may result from their unique GABAergic input being disinhibited via network effect. We conclude that dendritic spines receive specialized, diverse GABAergic inputs, and group III mGluRs differentially regulate GABAergic synaptic transmission to distinct GABAergic cell types in human cortex.

## Introduction

Dendritic spines receive the vast majority of synapses in the cerebral cortex and most of the presynaptic terminals release glutamate to them (Ramón y Cajal 1888; Kasthuri et al. 2015; Cano-Astorga et al. 2021). Dendritic spines are highly plastic structures and change their shape and size in response to synaptic activity (Maletic-Savatic et al. 1999; Holtmaat et al. 2006; Araya et al. 2014). Those connected to their parent dendritic shafts via narrow spine necks functionally isolate synaptic junctions from each other and allow independent changes in membrane potential (Kwon et al. 2017; Cornejo et al. 2022), receptor activity (Matsuzaki et al. 2001), signaling molecular mechanisms such as changes in calcium concentration (Yuste et al. 2000; Chen et al. 2011), and use-dependent changes in individual synaptic strengths (Matsuzaki et al. 2004; Lee et al. 2009). Most dendritic spines originate from pyramidal cells, in addition to other glutamatergic cortical neurons such as spiny stellate cells in layer IV, and receive a single synaptic input. With few exceptions, GABAergic neurons have fewer or no spines. A relatively small, but significant fraction of dendritic spines also receive an additional GABAergic synapse next to the glutamatergic one, as shown in rodents (Kubota et al. 2007), the cat (Beaulieu and Somogyi 1990), and monkey (Beaulieu et al. 1992). Because of the vast volume density of dendritic spines, GABAergic synapses on spines are more than twice as frequent as on cell bodies in both the monkey and cat cortex (Beaulieu et al. 1992).

The relative isolation of dendritic spine inputs from the rest on the neuron suggests that glutamatergic and GABAergic synapses on a single spine have more influence on each other than on other inputs to the same neuron. It has been shown that spine-innervating GABAergic synapses can selectively attenuate somatically and synaptically evoked calcium influx into single dendritic spines (Chiu et al. 2013). However, how this influences synaptic plasticity is unknown. It is also unknown how such doubly innervated spines are distributed on the neuron, or if the spines receiving a GABAergic input are just a random subset or a functionally distinct population. That the latter may be the case is indicated by the finding that in the rat frontal cortex, spines receiving a thalamo-cortical glutamatergic input are more likely to be innervated also by a GABAergic synapse than other spines (Kubota et al. 2007). Thus, cortical GABAergic neurons, which might specialize in spine innervation, could selectively regulate particular glutamatergic inputs to a given postsynaptic neuron.

Several distinct types of GABAergic cortical interneuron have been shown to innervate dendritic spines to differing degrees in addition to dendritic shafts and somata in rodents (Kawaguchi and Kubota 1998; Kubota et al. 2007), the cat (Somogyi et al. 1983; Kisvárday et al. 1985; Tamás et al. 1997a), and the monkey (Somogyi and Cowey 1981). There are clear molecular and some synaptic circuit homologies among the GABAergic cell types of the rodent and human cerebral cortex (Hodge et al. 2019; Bakken et al. 2021). However, with the exception of the GABAergic axo-axonic cell (Kisvárday et al. 1986), the basket cell (Szabadics et al. 2006), and the rosehip cell (Boldog et al. 2018) at present, it is unknown to what degree distinct human GABAergic neuronal types dedicate their outputs selectively to functionally different subcellular domains of postsynaptic neurons. A potential compartmentalization of the postsynaptic neuron surface by local GABAergic neurons has fundamental consequences for the regulation of multiple synaptic inputs to a given neuron and for the temporal dynamics of input to output transformation (Lovett-Barron et al. 2012; Somogyi et al. 2014; Doron et al. 2017).

In the context of synaptic plasticity and the GABAergic innervation of dendritic spines, a particular interest is the iconic double bouquet cell (DBC), which in the cat and monkey appears to innervate dendritic spines preferentially (Somogyi and Cowey 1981; Tamás et al. 1997a) and forms a system of radial GABAergic projection through cortical layers (Somogyi et al. 1981). In the current study, the name “DBC” is used in a narrow sense for neurons with cell bodies in layers 2 and upper 3 and having descending “horsetail”-like narrow axonal bundles through all layers. The “horsetail” axon of DBCs in primates strongly resembles the regularly spaced radial axonal bundles revealed by immunoreactions to the calcium binding protein calbindin (CB), which has been used to study these cells (DeFelipe et al. 1989; del Río and DeFelipe 1995; Peters and Sethares 1997). The small diameter radial axon bundle has been the subject of speculation regarding its potential roles in “minicolumns” (del Río and Defelipe 1997; DeFelipe et al. 2006), but the activity and cortical role of DBCs remains unknown.

Another iconic GABAergic cell type is the “basket cell”, so named because it is assumed that its terminals form perisomatic baskets that are thought to have a large inhibitory effect on the output of the postsynaptic neurons. Basket cells, including some parvalbumin-expressing (PV+) cells, show great diversity in molecular expression (Tasic et al. 2018; Hodge et al. 2019; Gouwens et al. 2020; Bakken et al. 2021), lateral spread and interlaminar specificity of their axons (Somogyi et al. 1983; DeFelipe et al. 1986; Kisvarday et al. 1987), and their synaptic output to different postsynaptic targets on the same cell, including dendritic spines (Somogyi et al. 1983; Somogyi and Soltész 1986; Kawaguchi and Kubota 1998; Szabadics et al. 2006). In the human cerebral cortex, the synaptic output selectivity, if any, of PV+ basket cells is unknown.

In the present study on the human cerebral cortex, we explored and compared the evoked firing behavior and synaptic connectivity of DBCs and putative basket cells in surgically resected samples, which were removed from patients for the treatment of tumors or temporal lobe epilepsy. We have also tested if synaptic inputs to the 2 distinct cell types differed in their regulation by glutamate receptors. We chose to study group III metabotropic glutamate receptors (mGluRs), because of previous indications from studies on rodents that group III mGluRs are differentially expressed in cortical GABAergic neurons (Dalezios et al. 2002; Somogyi et al. 2003) and selectively regulate GABAergic synaptic transmission (Semyanov and Kullmann 2000; Kogo et al. 2004; Klar et al. 2015), and because of the large investment into developing drugs acting on group III mGluRs for the treatment of neurological and psychiatric conditions (Maksymetz et al. 2017; Charvin 2018; Ferraguti 2018). The results presented here, some of which have been published in abstract form (Lukacs et al. 2019, 2020), reveal a high degree of multidimensional specialization of human GABAergic interneurons.

## Materials and methods

### Ethical approval and patient consent

Human tissue samples were collected from patients undergoing neurosurgery at the John Radcliffe Hospital (Oxford) for the treatment of brain tumors or temporal lobe epilepsy (Table 2) in accordance with the Human tissue Act 2004 (UK), under the ethical license (15/SC/0639) of the Oxford Brain Bank (OBB), Department of Neuropathology, John Radcliffe Hospital, Oxford, UK. Patients consented to providing the samples after they were fully informed by a medical professional. All collected samples represented “access tissue,” which otherwise would have been discarded, that is tissue necessarily removed in order to access the diseased part of the brain, but not or only partially affected by the pathological process. An additional 3 tissue samples (see Table 2) were imported through the OBB from the laboratory of Gábor Tamás, at the University of Szeged, Hungary, which were collected after informed consent of patients in the Department of Neurosurgery, University of Szeged, Hungary, under the ethical license (75/2004), as specified by Hungarian law and the University of Szeged.

### Sample collection and slice preparation

Samples were collected between 10 AM and 5 PM (UK time) as described previously (Bocchio et al. 2019; Field et al. 2021). A small (0.5–2 cm^3^) block of neocortex was dropped into ice-cold cutting artificial cerebrospinal fluid (ACSF) saturated with carbogen (95% O_2_, 5% CO_2_) and divided into smaller blocks as necessary. The cutting ACSF contained the following (in mM): 65 sucrose, 85 NaCl, 25 NaHCO_3_, 2.5 KCl, 1.25 NaH_2_PO_4_, 0.5 CaCl_2_, 7 MgCl_2_, 10 glucose (pH ~ 7.3; ~300 mOsm/L) for samples A-C and AA-CC (see Table 2), or the following (in mM): 92 N-methyl-D-glucamine (NMDG), 2.5 KCl, 1.25 NaH2PO4, 30 NaHCO_3_, 20 4-(2-hydroxyethyl)-1-piperazineethanesulfonic acid (HEPES), 25 glucose, 2 thiourea, 5 Na-ascorbate, 3 Na-pyruvate, 0.5 CaCl_2_, and 10 MgSO_4_ (pH ~ 7.3, ~300 mOsm/L) (Ting et al. 2018) for the remainder. Samples were transported over 15–20 min to the laboratory in a sealed transport bottle, at ~4 °C. Slices of ~350-μm thickness were prepared as described in Field et al. (2021), except for samples A-C, the cutting ACSF was gradually replaced by recording ACSF, in which they were stored at room temperature until recording. The recording ACSF contained the following (in mM): 130 NaCl, 3.5 KCl, 1.3 NaH_2_PO_4_, 24 NaHCO_3_, 3 CaCl_2_, 1.5 MgSO_4_, and 12.5 glucose (pH ~ 7.3, ~300 mOsm/L). The storing ACSF contained the same components as the NMDG-based cutting ACSF except the NMDG was replaced with 92 mM NaCl. All solutions were continuously bubbled with carbogen.

### Electrophysiological recordings

Electrophysiological recordings were performed over 10–16 h after slicing between 12 PM and 4 AM the next day as described previously (Bocchio et al. 2019; Field et al. 2021). Slices in the recording chamber were perfused continuously with recording ACSF saturated with carbogen at a flow rate of ~10 mL/min using a peristaltic pump (Gilson). The time required for a new solution to reach the chamber was 45–60 s. Glass capillaries (4–7 MΩ) were filled with an internal solution containing the following (in mM): 126 K-gluconate, 4 KCl, 4 ATP-Mg, 0.3 GTP-Na_2_, 10 Na_2_-phosphocreatine, 10 HEPES, 0.03 ethylene glycol-bis(2-aminoethylether)-N,N,N′,N′-tetraacetic acid (EGTA), and 0.05% (w/v) biocytin (pH ~ 7.3, 280–290 mOsmol/L). Neurons were visualized by differential interference contrast (DIC) microscopy with an Olympus BX51WI microscope equipped with a LUMPlanFL 60x water immersion objective (Olympus) and a digital camera (Zyla, ANDOR), connected to a desktop computer. Whole-cell patch-clamp recordings were performed from neurons in layers II and III, at 33–37 °C, using either an EPC-10 triple patch clamp amplifier with Patchmaster software (HEKA) or a Multiclamp 700B amplifier with pClamp software (Molecular Devices). Data were digitized at 100 kHz for current-clamp recordings and at 50 kHz for voltage-clamp recordings on the EPC-10 amplifier; on the Multiclamp amplifier, data were digitized at 10 kHz in both modes. The reported voltage values are not compensated for a 16.5-mV junction potential. For each cell, voltage responses were recorded to a series of 800-ms-long current square pulses starting from holding current −100 pA until rheobase (RB) +100 pA with 20 pA increments between sweeps in current clamp mode (I–V traces). The initial holding current was between 0 and −100 pA and was aimed to be a multiple of 20 pA. Bridge balance was not adjusted during current clamp recordings. In paired recordings, a pair or a train of 5 action potentials (APs) were evoked at 50-ms intervals in 1 neuron by brief current injections, while the other neuron was continuously held at just subthreshold membrane potentials (−40 to −45 mV, E_Cl_- ≈ −94 mV) in voltage-clamp mode in order to detect GABA_A_ receptor-mediated unitary-evoked inhibitory postsynaptic currents (eIPSCs). For pair No-2, a test presynaptic AP was evoked 1 s after the last AP in the previous train (see Fig. 3d). Spontaneous inhibitory postsynaptic currents (sIPSCs) were recorded by continuously clamping the neurons to just subthreshold membrane potentials (−40 to −45 mV). After recording a stable baseline for 3–5 min, group III mGluRs were activated by bath application of the orthosteric agonist, L-2-amino-4-phosphonobutyric acid (L-AP4, 50 or 300 μM). At the lower concentration, L-AP4 is thought to activate only the high affinity mGluR subtypes, 4 and 8, whereas at the higher concentration, it would also activate mGluR7 (Cartmell and Schoepp 2000; Kogo et al. 2004). Finally, the drug was allowed to wash out for 5–15 min, by perfusion of recording ACSF. Uncompensated series resistance was monitored every minute by application of a 10-ms voltage step of −10 mV. APs and ionotropic glutamate receptors were not blocked.

In order to test the nature of the spontaneous synaptic currents recorded under our conditions, we recorded 6 interneurons and 2 pyramidal cells in whole cell voltage clamp mode held at −50 mV membrane potential with an E_Cl_ = −94 mV. Four of the interneurons were visualized and they were all confirmed to be interneurons with cell bodies in layer II. Two had sparsely spiny dendrites, whereas the dendrites of the other 2 were smooth and beaded. Two of the interneurons showed immunoreactivity to cannabinoid type 1 receptor (CB1) along their axonal membranes, one of them being immunoreactive also for cholecystokinin (CCK) in the cell body. Further characteristics of these cells are not reported further in this study. Bath application of the GABA_A_ receptor antagonist gabazine (16 μM) completely abolished sIPSCs recorded in these interneurons and pyramidal cells, while sEPSCs remained unaffected (Supplementary Fig. 1a). In an additional pair of visualized and synaptically connected interneurons located in layers III, gabazine blocked the eIPSCs (Supplementary Fig. 1b). The presynaptic interneuron was a bitufted neuron with long, radially running, smooth dendrites, and dense local axon, and the postsynaptic interneuron was a multipolar smooth-dendritic cell, whose axon was not recovered. The presynaptic neuron was tested for CB1 in the axon and was immunonegative, and both cells were tested for VIP in their soma and both were immunonegative. The effect of gabazine is consistent with the prediction that spontaneous and evoked unitary IPSCs recorded under our experimental conditions were mediated by GABA_A_ receptors.

### Data analysis and inclusion criteria

The following criteria were applied for inclusion of cells in the analysis: (i) the holding current was between 0 and −100 pA for holding the cell at −75 mV; (ii) at least, one overshooting AP could be elicited by depolarising current injections; and (iii) fast pipette capacitance was successfully compensated and no oscillation artifacts were observed in current clamp mode. The following additional criteria were applied for inclusion of the voltage clamp recordings of eIPSCs and sIPSCs: (i) series resistance (Rs), calculated as the ratio of the −10 mV voltage step and the peak capacitive current transient, was <35 MΩ during the entire recording and did not change more than 25% from the initial value, and (ii) there were no sudden shifts in Rs.

PatchMaster (.dat) files were opened in Igor Pro software v7.0.8.1 (WaveMetrics) using Patchers’s Power Tools (Department of Membrane Biophysics, Max Planck Institute for Biophysical Chemistry, Göttingen, Germany^1^). After digitally adjusting the I–V traces to remove the artifact resulting from the bridge balance, the following parameters were measured: input resistance (IR) was calculated as the slope of the linear fit to subthreshold steady state membrane voltage values and the injected current (*V* = *a* + b*I, where *V* is the steady state membrane voltage, *I* is the injected current, and *b* is the IR). Resting membrane potential (Vm) was measured as the steady state voltage in response to 0-pA current injection or was calculated as the constant “a” in the formula above. The membrane time constant ($\tau$) and whole-cell capacitance (C_m_) were calculated from current-clamp recordings as described (Golowasch et al. 2009): $\tau$ was calculated as the time constant of the slowest component of a double-exponential fit to the voltage in response to holding current −100-pA current injection from the beginning of the step until the peak negative deflection (Vm(t) = *V*_0_ + $\sum_{i=1}^2{V}_i{e}^{-t/\tau }$, where *V*_0_ is the baseline membrane potential and *V_i_* and $\tau$ are the amplitude and the time constant of each exponential term); C_m_ was obtained by dividing $\tau$ with the amplitude of the voltage deflection associated to the slowest exponential term. The sag ratio was calculated as the ratio of the maximum and the steady state voltage deflection in response to holding current −100-pA current injection. The rheobase was measured as the current injected evoking the first AP. Single AP kinetic parameters were measured on the first AP elicited at RB current injection in Matlab R2020a (MathWorks), using a custom written script as described previously (Field et al. 2021).

Voltage-clamp recordings were filtered with a 2-kHz lowpass Bessel (8-pole) filter and boxcar smoothing (11 points) in Clampfit or IgorPro before analysis. Evoked IPSCs were detected with TaroTools toolbox for Igor Pro (https://sites.google.com/site/tarotoolsregister), using a threshold (4–7 pA) detection method, followed by visual inspection. Trials in which no eIPSC was detected were considered as failures. The response lag was measured from the peak of the presynaptic AP to the onset of the eIPSC. The mean amplitude of eIPSCs was calculated from all trials, including failures. The paired-pulse ratio, an indicator of presynaptic release probability, was calculated as the mean amplitude of IPSC2 over IPSC1. The half-width and the risetime (20–80%) were calculated automatically by the detection software. Spontaneous IPSCs were analyzed in Clampfit v10.7.0.3 (Molecular Devices) from all 3–5 min of baseline and the last 2 min of drug application to allow for the drug to reach maximum concentration in the recording chamber and achieve reasonable penetration into the slice. Washout periods were not analyzed due to instability. For the detection of sIPSCs, first, a typical IPSC was visually identified in the baseline period and was used as a template for automatic detection. Events with an amplitude smaller than 5 pA and a risetime (20–80%) longer than 5 ms were removed, and the remaining events were averaged to create a second template, which was then used in a second round of event detection. Control recordings for testing potential changes in sIPSCs over time were recorded alternately with test recordings during every session by perfusion of vehicle (recording ACSF) for 15–20 min.

All statistical tests were carried out in Origin Pro (v 94E 2017, OriginLab) and are reported in full in the results. Values are reported as mean ± standard deviation, unless stated otherwise. Normality of the data was tested with Kolmogorov–Smirnov test, and the appropriate parametric or nonparametric tests were applied. For data with <10 observations, a nonparametric test was used. Differences were considered statistically different at alpha <0.05.

### Biocytin visualization and immunohistochemistry

After completion of the electrophysiological recording, slices were immersed in fixative, containing 4% (w/v) paraformaldehyde (PFA) and 15% (v/v) saturated picric acid (PA) in 0.1 M PB at pH ~ 7.2 at 4 °C overnight. For samples from cases O-W (see Table 2), the fixative also contained 0.05% (w/v) glutaraldehyde (GA). Next, slices were thoroughly washed and resectioned into 3–5, ~60-μm-thick sections on a vibratome as described previously (Field et al. 2021). From each slice, 2 sections, including the one in which the soma was predicted to be found, were selected for fluorescent visualization. Sections were visualized using Alexa 488-conjugated streptavidin (1:1,000) as described previously (Bocchio et al. 2019; Field et al. 2021).

The expression of multiple molecules in labeled cells and in control tissue was tested using immunohistochemical reactions on single 60- to 70-μm-thick sections. Immunohistochemical reactions were performed as described previously (Unal et al. 2015; Joshi et al. 2017). Up to 5 different primary antibodies (ab), produced in different host species, against the epitopes of the molecules of interest were used in appropriate dilutions on any single section. For a list of all primary abs, see Table 1. Secondary abs against immunoglobulin G of the host species of the primary ab, conjugated to different fluorophores, were added at appropriate dilutions (Alexa405/DyLight405 (blue)- and cyanine5 (Cy5)/DyLight649 (infra-red)-conjugated abs at 1:250; Alexa488 (green)-conjugated abs at 1:1,000; Cy3 (red)-conjugated abs at 1:400). All secondary abs were raised in donkey (Jackson Immuno Research).

**Table 1:** Primary antibody information.

Molecule	Host species	Antigen	Clonality	Internal ref.	Dilution	Stock protein concentration (μg/mL)
calbindin	rabbit	recombinant rat calbindin D-28 k	polyclonal	989	1:5,000	antiserum
calbindin	goat	mouse calbindin D-28 k (NM009788) expressed in bacteria	polyclonal	1427	1:500	200
cannabinoid type 1 receptor (CB1)	guinea-pig	mouse CB1, C-terminal 31 aa (NM007726)	polyclonal	1274	1:5,000	200
CB1	rabbit	synthetic peptide aa residues 443–473, mouse and human	polyclonal	1558	1:10,000	1
cholecystokinin (CCK)	mouse	gastrin-17	monoclonal	1052	1:1,000	5000
calretinin (CR)	goat	human recombinant calretinin	polyclonal	1116	1:1,000	antiserum
hexaribonucleotid binding protein-3 (NeuN)	mouse	-	monoclonal	842	1:1,000	1000
parvalbumin (PV)	guinea-pig	rat recombinant protein aa 1–110	polyclonal	1310	1:1,000–1:5,000	antiserum
PV	goat	rat muscle parvalbumin	polyclonal	1258	1:1,000	antiserum
PV	mouse	purified carp II parvalbumin	monoclonal	922	1:1,000	ascites
somatostatin (SM)	sheep	somatostatin conjugated to carrier protein	polyclonal	1085	1:500–1:5,000	5000
SM	mouse	somatostatin-14 conjugated to carrier protein	monoclonal	1276	1:400	140
vesicular GABA transporter (VGAT)	guinea-pig	rat recombinant protein aa 2–115	polyclonal	1321	1:500–1:1,000	antiserum
vasoactive intestinal polypeptide (VIP)	rabbit	porcine VIP coupled to bovine thyroglobulin with carbodiimide linker	polyclonal	1523	1:5,000	antiserum
VIP	mouse	VIP	monoclonal	1053	1:25,000	antiserum
γ-aminobutyric acid (GABA)	rabbit	GABA-bovine serum albumin conjugate	polyclonal	9	1:1,000	antiserum
Molecule	Host species	Source	Product code	Specificity reference		
calbindin	rabbit	SWANT	CB-38	(Airaksinen et al. 1997)		
calbindin	goat	Frontier Institute	Calbindin-Go-Af1040	(Nakagawa et al. 1998)		
cannabinoid type 1 receptor (CB1)	guinea-pig	Frontiers Institute	CB1-GP-Af530	(Fukudome et al. 2004)		
CB1	rabbit	Immunogenes	Anti-CB1 (Rabbit) Polyclonal Antibody	(Dudok et al. 2015)		
cholecystokinin (CCK)	mouse	CURE/Digestive Diseases research Center, Antibody/RIA Core, NIH Grant #DK41301	9303	(Ohning et al. 1996; Geerling et al. 2006)		
calretinin (CR)	goat	SWANT	CG1	(Schiffmann et al. 1999)		
hexaribonucleotid binding protein-3 (NeuN)	mouse	Chemicon International	MAB377	(Tippett et al. 2007)		
parvalbumin (PV)	guinea-pig	Synaptic Systems	195,004	Manufacturer’s spec.		
PV	goat	SWANT	PVG-213	(Schwaller et al. 1999)		
PV	mouse	SWANT	235	(Celio et al. 1988)		
somatostatin (SM)	sheep	Cortex Biochem	CR2056SP			
SM	mouse	Genetex	GTX71935	(Kubota et al. 2011)		
VGAT	guinea-pig	Synaptic Systems	131,004	(Ramer 2008)		
VIP	rabbit	Immunostar	20,077	Manufacturer’s spec.		
VIP	mouse	CURE/Digestive Diseases research Center, Antibody/RIA Core, NIH Grant #DK41301	55	(Wong et al. 1996)		
GABA	rabbit	raised in house	n.a.	(Hodgson et al. 1985)		

### Wide field epifluorescence and confocal laser scanning microscopy

**Fig. 1:**
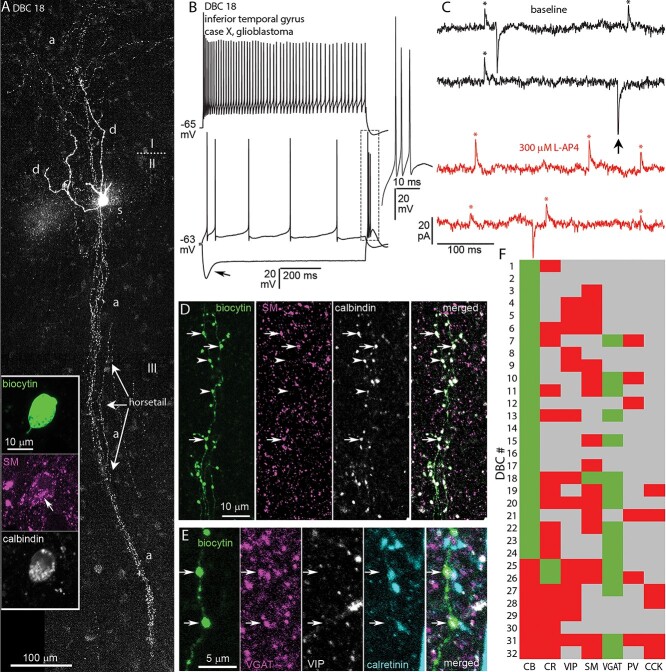
In vitro recorded and biocytin labeled DBC in the human inferotemporal neocortex. a) Tiled confocal fluorescent image of the cell with descending columnar “horsetail” axon. The cell body (s) is located in layer II with dendrites (d) penetrating layer I. The axon (a) diffusely innervates layer I (Z stack of 50 optical slices of 1.8-μm thickness, total depth, 45.9 μm); roman numbers indicate layers; inset: the cell body showing immunoreactivity for SM (Golgi apparatus, magenta, arrow) and CB (white) in the cytoplasm (Z stack of 5 optical slices of 0.9-μm thickness, total depth, 2.4 μm). b) Voltage responses of the cell in A to current injections of rheobase (RB), RB +100, and holding current −100 pA, showing a voltage sag (arrow) and rebound spikes (boxed area, inset) in response to hyperpolarizing current injection and firing with spike frequency adaptation and strong spike amplitude accommodation when continuously depolarized; inset showing the expanded rebound burst from the boxed area. c) Example current traces recorded in voltage-clamp mode at −50 mV clamping potential, showing spontaneous inhibitory (asterisks, upward deflections, sIPSC) and excitatory (arrow, downward deflections, sEPSC) postsynaptic currents in baseline condition (black) and after bath application of 300 μM L-AP4 (red). d) Boutons of the cell in A are immunopositive for SM (magenta, arrows) and CB (white); note that some boutons are immunonegative for somatostatin (arrowheads) (Z stack of 3 optical slices of 0.9-μm thickness, total depth, 1.8 μm). e) Boutons (arrows) of the cell in A are immunopositive for VGAT (magenta), and immunonegative for VIP (white) and CR (cyan) (Z stack of 3 optical slices of 0.9-μm thickness, total depth, 1.8 μm). f) Immunoreactivities in all recovered and tested neurons with descending axon bundles: green, immunopositive; red, immunonegative; gray, not tested/inconclusive.

Cells visualized with Alexa 488-conjugated streptavidin and fluorescent immunoreactions were first evaluated in a wide field epifluorescence microscope (Leitz DMRB, Leica) using PL FLuotar 20x/0.5 and 40x/0.7 lenses and photographed with an ORCA ER digital camera (Hamamatsu Photonics) controlled by OpenLab software (Improvision). The light source was either a pE-300 LED lamp (CoolLED) or a mercury arc lamp (HBO, Osram), for which the light was spectrally separated by dichroic mirrors to obtain optimal excitation and emission bandwidths for each fluorophore. For higher resolution imaging, confocal laser scanning microscopy was performed using an LSM 710 axioImager.Z1 microscope (Zeiss) and DIC M27 Plan-Apochromat 40x/1.3, 63x/1.4, and alpha Plan-Apochromat 100x/1.46 oil immersion objective lenses, controlled by ZEN 2008 software (v 5, Zeiss), as described previously in detail (Lasztóczi et al. 2011). Signal from each fluorophore was recorded in separate scanning tracks and channels, using the following lasers: for Alexa405 and DyLight405, a 405-nm solid state laser; for Alexa488, a 488-nm argon laser; for Cy3, a 543-nm He-Ne laser; for Cy5 and DyLight647, a 633 He-Ne laser. Pinhole size was adjusted optimally for similar optical slice thickness between tracks. The step size along the Z imaging axis was set to half of the thickness of the optical slices, as per the Nyquist criterion for optimal sampling. Details of the optical slice thickness are given in the figure descriptions. Some neurons were imaged in the entire ~60- μm-thick section to demonstrate the distribution of their dendrites and axon (see Fig. 1a). In order to remove the autofluorescence signal of the lipofuscin, these sections were imaged in a spectral scanning mode (λ-stack mode in ZEN software) with a fully opened pinhole, after which regions of interest including the labeled processes of neurons, lipofuscin, and the background, respectively, were selected to determine the emission spectra of each. This was used to separate the signals into different pseudo-channels by spectral linear unmixing (Dickinson et al. 2001), which was performed in the ZEN software.

### Peroxidase reactions

Sections of labeled cells used for immunohistochemistry were ultimately converted by avidin-biotin horseradish peroxidase (HRP) reaction with 3,3′-diaminobenzidine (DAB) as chromogen for the visualization of biocytin and embedded in epoxy resin (Durcupan, Sigma) for conventional light microscopic examination. Sections were incubated in biotinylated peroxidase complex (B) 1:100 v/v (Vectastatin ABC elite kit, Vector Laboratories) in TBS-Tx for 4 h at room temperature, then incubated in avidin + B (A + B)1:100 v/v in TBS-Tx over 36 h at 4 °C. Next, sections were pre-incubated in 0.5 mg/mL DAB in Tris Buffer (TB) for 10 min in dark. Subsequently, H_2_O_2_ substrate was added to a final concentration of 0.002% w/v to initiate DAB polymerization. After 12–20 min, depending on the intensity of the labeling, reactions were stopped and sections were washed 4 × 10 min in 0.1 M PB. For contrast enhancement, sections were incubated in 0.5% w/v Osmium tetroxide solution in 0.1 M PB for 1 h at room temperature. Before mounting, sections were dehydrated using increasing concentrations of ethanol and final steps of propylene oxide (Sigma) for 2 × 10 min. From propylene oxide, sections were quickly transferred into Durcupan epoxy resin (Sigma) and left overnight and finally mounted on glass slides.

For electron microscopy, sections of slices fixed without GA were postfixed with same fixative as above with the addition of GA to a final concentration of 0.05% (w/v) for 2 h at 4 °C. Sections were then incubated in 20% sucrose (Sigma Aldrich) in 0.1 M PB for 2 h at room temperature for cryoprotection, followed by quickly freezing the sections using liquid nitrogen and thawing them in PB with sucrose (1–3 times). All following steps were the same as for HRP reactions on Triton-treated sections with the exception that the buffer did not contain detergent. During dehydration, these sections were treated with 1% (w/v) uranyl acetate (UA) dissolved in 70% ethanol for 40 min in dark for further enhancement of contrast for electron-microscopy.

### Light microscopy and reconstruction of labeled cells

Transmitted light microscopy of HRP-converted sections was performed using a Leitz Dialux22 microscope (Leica), equipped with NPL Fluotar 10x/0.3, 25x/0.55, 40x/0.78 dry, and PL Apo 100x/1.32 oil immersion objective lenses. Images were taken using a Canon EOS 40D digital camera controlled by EOS Canon Utility software. Some neurons were reconstructed from multiple sections in order to demonstrate the distribution of their dendrites and axon across different neocortical layers, as well as for the identification of their axonal boutons, which were later examined using electron-microscopy. For 2D reconstruction of labeled cells (see Figs. 4a and 7a), neurons were manually traced using a drawing tube attached to a transmitted light microscope (Leitz Dialux22, Leica), equipped with a Pl Apo 63x/1.4 oil immersion objective. The India ink drawings were digitized using a digital camera (EOS 40D, Canon) and processed in Adobe Photoshop. Digital 3D reconstruction of some neurons (see Fig. 6a) was performed with Neurolucida software (v 2020 February 2 MBF Bioscience) and an Axioskop 2 mot plus (Zeiss) equipped with a Plan Apochromat 100x/1.4 Oil lens and Retiga 2000R (QImaging) digital camera, or with a v 2021 January 3 software and a Leica DMR microscope equipped with a HCX PL Fluotar 100x/1.30 Oil lens and Lumina HR (MBF Bioscience) digital camera.

### Serial section transmission electron microscopy of labeled cells and reconstruction of target dendrites

After the labeled neurons were reconstructed, some were selected for electron-microscopic study of their synapses (see Table 3 and Fig. 6). Areas of the HRP-converted sections containing the “horsetail” axon of DBCs in layer III were removed from the glass slides and re-embedded in cylindrical blocks of epoxy resin. Electron-microscopic sections (~50 nm) were cut using an Ultra 45 Diatome diamond knife (TAAB) and mounted on pioloform-coated copper slot grids (2 × 1 mm, 10–20 sections/grid). Grids were examined using either a Fei Tecnai 12 transmission electron microscope (TEM) at 120-keV accelerating voltage and photographed with a Gatan OneView CMOS camera controlled by Gatan Digital Micrograph software (GMS3), or using a Jeol-1011 microscope at 80-keV accelerating voltage and photographed with a TRS Sharp Eye camera controlled by iTEM software. The labeled processes and their synaptic targets were photographed in serial electron microscopic sections, were imported into TrackEM2 reconstruction plugin of ImageJ software, and were aligned using an automatic nonlinear alignment method, based on block correspondence. After aligning the axon and boutons of the labeled cell, the target structure and the synaptic specialization were traced through the serial sections and were rendered into a 3D reconstruction (see Fig. 6k and l).

### Postembedding immunohistochemistry for GABA and counting of synapses

Samples AA-CC were fixed with 4% (w/v) PFA, 2.5% (w/v) GA, and 15% (v/v) PA and were used for postembedding immunohistochemistry for GABA. Sections of these samples were treated with 1% (w/v) Osmium tetroxide, 1% (w/v) UA, and mounted on glass slides in epoxy resin (Durcupan). From each sample, an area of layer III was re-embedded for electron microscopy. Serial sections of ~60-nm thickness were mounted on pioloform-coated nickel slot grids (2 × 1 mm). Postembedding immunohistochemistry was performed as described (Somogyi and Soltész 1986) using rabbit antiserum to GABA at 1:1,000 dilution (Hodgson et al. 1985) and 6-nm gold-coupled anti rabbit IgG (Aurion) as secondary antibody. Silver enhancement was used for easier detection of the particles in electron micrographs as recommended by the manufacturer (Aurion).

For counting synapses, electron micrographs of serial immunoreacted sections (5–10) were taken with a Philips CM100 TEM equipped with a Gatan UltraScan 1000 CCD camera at 80-keV accelerating voltage, and 4, 5, and 2 different areas were photographed from samples AA, BB, and CC, respectively, chosen not to contain somata or blood vessels. Electron micrographs were imported in trackEM2 software and serial sections were aligned as described above. Synapses were counted using a stereological method, by tracing the pre-and postsynaptic elements in 3D through a specified volume within a 3D counting frame (West 1999; Cano-Astorga et al. 2021). The first section and the top and left sides formed the exclusion planes for any synapse, whereas the last section and the bottom and right sides formed the inclusion planes. Synapses were excluded if the synaptic specialization in the pre- or postsynaptic membrane (see Fig. 8) touched any of the exclusion planes. The volumes of the counting frames were corrected by a factor of 1.23 compared with the scale of the micrographs to compensate for compression along the direction of cutting of the electron microscopic sections. This was calculated by measuring in the direction of cutting the block surface width and the section width after cutting. Thus, the calculated density of synapses is given for fixed, osmium-treated and dehydrated specimens, not the living brain.

**Table 2:** Patient information.

Patient code	Sex	Age range	Cells	Cortical region	Diagnosis (type)	Cortical infiltration	Intracranial pressure/oedema
A	M	50–54	PV-DTC 14	rITG	Diffuse glioma (WT)	Not known	Not known
B	F	40–44	DBC 1	rMFG	Glioblastoma (IDH1 mutant)	No	No
C	F	55–59	DBC 2,3	rITG	Metastatic melanoma (WT)	No	No
D	M	65–69	DBC 27,28 GBZ 1, 2	rSPL	Glioblastoma (WT)	NC	No
E	M	50–54	PV-DTC 8	rF	Glioblastoma (WT)	Yes	Yes
F	F	18–20	DBC 4, 5, 6	rMTG	Focal epilepsy (n.a.)	No	No
G	M	80–84	DBC 29	rT	Glioblastoma (WT)	Yes	Yes
H	M	60–64	DBC 7, 8, 9	POL	Metastatic adenocarcinoma (WT)	Yes	Yes
I	F	65–69	DBC 10,30 PV-DTC 12	rT	Glioblastoma (WT)	NC	Yes
J	M	50–54	DBC 11, 12	rP	Glioblastoma (WT)	NC	Yes
K	F	70–74	DBC 13 CTR 1	rSFG	Metastatic melanoma (WT)	No	No
L	F	60–64	CTR 2	rITG	Glioblastoma (WT)	Yes	Yes
M	M	40–44	DBC 14	rSTG	Oligodendroglioma (WT)	Yes	No
N	M	50–54	DBC 15	rITG	TLE (n.a)	No	No
O	M	40–44	PV-DTC 13	rITG	Oligodendroglioma (WT	Yes	Yes
P	M	60–64	PV-DTC 1 CTR 3	rMTG	Glioblastoma (WT)	NC	Yes
Q	F	30–34	PV-DTC 5	lMFG	Astrocytoma (IDH1 mutant)	Yes	No
R	F	55–59	PV-DTC 2	rMTG	Meningioma (n.a.)	NC	No
S	F	60–64	DBC 16, 17 CTR 4,5	lMFG	Glioblastoma (WT)	NC	No
T	M	60–64	PV-DTC 3, 4, 6, 7	lMTG	Glioblastoma (WT)	NC	Yes
U	M	50–54	CTR 6	rITG	Glioblastoma (WT)	No	No
V	F	35–39	PV-DTC 9	rITG	Anaplastic astrocytoma (IDH1 mutant)	NC	Yes
X	F	50–54	DBC 18, 31 PV-DTC 10, 11 CTR 7	lITG	Glioblastoma (WT)	Yes	No
Y	M	60–64	DBC 19, 20, 25, 32	rIPL	Dysembryoplastic neuroepithelial tumor (n.a.)	No	No
Z	M	60–64	DBC 26	rITG	Anaplastic astrocytoma (IDH1 mutant)	No	No
W	F	55–59	DBC 21, 22, 23, 24	rITG	Anaplastic astrocytoma (IDH1 mutant)	NC	No
AA	F	55–59	n.a.	rT	Subarachnoid hemorrhage (n.a.)	n.a.	Not known
BB	F	45–49	n.a.	rF	Subarachnoid hemorrhage (n.a.)	n.a.	Not known
CC	M	55–59	n.a.	rF	Metastatic tumor (not known)	Not known	Not known
DD	M	50–54	GBZ 3, 4, 9, 10	lSFG	Subependymoma	No	No
EE	M	40–44	GBZ 5, 6	F	Glioblastoma (WT)	Not known	Yes
FF	M	70–74	GBZ 7, 8	rMTG	Glioblastoma (WT)	Not known	Yes

Samples AA—CC were obtained at and imported from the University of Szeged, Hungary; ITG—inferior temporal gyrus; STG—superior temporal gyrus; MFG—middle frontal gyrus; SFG—superior frontal gyrus; SPL—superior parietal lobule; IPL—inferior parietal lobule; POL—parieto-occipital lobe; T—temporal; F—frontal; P—parietal; l—left; r—right; CTR—pharmacological control; GBZ—gabazine test; WT—wild type; IDH—isocitrate dehydrogenase 1; N.C.—not conclusive.

**Table:** 

Patient code	Seizures (onset)		Anticonvulsive medication	Steroid medication^*^	Other medication	Medical history	
A	Partial (not known)		Carbamazepine; levetiracetam	None	None	Posterior fossa astrocytoma; radiotherapy; traumatic brain injury; left temporal meningioma	
B	No (n.a.)		Levetiracetam	Dexamethasone	Omeprazole; sertraline	Depression	
C	No (n.a.)		None	Dexamethasone	None	Melanoma surgically removed	
D	No (n.a.)		None	Dexamethasone	Amlodipine; lansoprazole; atorvastatin	HTN	
E	Yes (not known)		Levetiracetam	Dexamethasone	Nicotine; omeprazole; lactulose	None	
F	Yes (2.5y)		Levetiracetam; lacosamide; carbamazepine	Dexamethasone	Depo injection contraceptive	None	
G	Yes (not known)		Levetiracetam	Dexamethasone	Metformin; salbutamol	Asthma (mild); type II DM	
H	No (n.a.)		Levetiracetam	Dexamethasone	None	Metastatic lung cancer; AKI	
I	No (n.a.)		None	Dexamethasone	Amlodipine	HTN	
J	No (n.a.)		Levetiracetam	Dexamethasone	None	None	
K	Yes (not known)		Levetiracetam	Dexamethasone	Lansoprazole	None	
L	No (n.a.)		None	None	Amlodipine	HTN	
M	Yes (3y)		Lamotrigine	Dexamethasone	None	None	
N	Yes (5y)		Carbamazepine; clobazam; lamotrigine; levetiracetam	None	Beclometasone inhaler	Asthma	
O	Yes (5y)		Levetiracetam	Dexamethasone	Hydrocoison; mirtazapine; testosterone; paracetamol; adcal-D3; levothyroxine	Secondary Addison’s	
P	No (n.a.)		Levetiracetam	Dexamethasone	Amlodipine; loratadine; paracetamol	HTN	
Q	Yes (4 m)		Levetiracetam	None	Beclometasone; salbutamol	Asthma	
R	Yes (2 m)		Levetiracetam	None	None	None	
S	No (n.a.)		None	Dexamethasone	Atorvastatin	High cholesterol	
T	Yes (15d)		Levetiracetam	Dexamethasone	None	None	
U	No (n.a.)		Levetiracetam	Dexamethasone	None	None	
V	Yes (10d)		Levetiracetam	Dexamethasone	None	None	
X	Yes (2 m)		Levetiracetam	None	Doxycycline	None	
Y	No (n.a.)		None	Dexamethasone	None	None	
Z	Yes (6y)		Levetiracetam	Dexamethasone	Amplodipine; atrovastatin; gliclazide; indapamide; metformine; perindopril	HTN; diabetes; high cholesterol	
W	No (n.a.)		None	None	Amlodipine; ramipril; sertraline	Depression, HTN	
AA	Not known		Not known	Not known	Not known	Not known	
BB	Not known		Not known	Not known	Not known	Not known	
CC	Not known		Not known	Not known	Not known	Not known	
DD	No (n.a.)		None	None	None	None	
EE	No (n.a.)		Levetiracetam	Dexamethasone	Omeprazole	Anxiety disorder, substance abuse	
FF	No (n.a.)		Levetiracetam	Dexamethasone	Atrovastatin; levemir; omeprazole; warfarin; salbutamol; insulin; beclometasone inhaler	Colorectal cancer; type I DM; peripheral vascular disease; DVT; asthma; thrombophlebitis	

*Refers to chronic steroid medication. All patients received dexamethasone perioperatively; DM – diabetes mellitus; DVT – deep venous thrombosis.

**Table 3:** Information on analyzed cells.

Cell #	Patient	Cell code	Data collected	
			Anatomy	Electrophysiology
DBC 1	B	JR180119_3_IL6	reconstruction, IHC	*I*–*V*
DBC 2	C	JR180425_6_IL2	IHC	*I*–*V*; eIPSC (pair1)
DBC 3	C	JR180425_7_IL1	IHC	*I*–*V*
DBC 4	F	JR190220_7_IL3	reconstruction; IHC	*I*–*V*
DBC 5	F	JR190220_7_IL4	reconstruction; EM; IHC	*I*–*V*
DBC 6	F	JR190220_8_IL1	IHC	*I*–*V*; eIPSC (pair2)
DBC 7	H	JR190410_1_IL4	IHC	*I*–*V*
DBC 8	H	JR190410_2_IL5	IHC	-
DBC 9	H	JR190410_3_IL1	IHC	*I*–*V*
DBC 10	I	JR190424_5_IL4	EM; IHC	*I*–*V*; sIPSC (L-AP4)
DBC 11	J	JR190426_3_IL3	IHC	*I*–*V*
DBC 12	J	JR190426_7_IL3	IHC	*I*–*V*; eIPSC (pair3)
DBC 13	K	JR190515_3_IL3	IHC	*I*–*V*
DBC 14	M	JR190628_1_IL2	IHC	*I*–*V*
DBC 15	N	JR190731_9_IL2	IHC	*I*–*V*
DBC 16	S	JR191030_1_IL1	IHC	*I*–*V*
DBC 17	S	JR191030_5_IL1	IHC	*I*–*V*; sIPSC (L-AP4)
DBC 18	X	JR200115_5_IL1	IHC	*I*–*V*; sIPSC (L-AP4)
DBC 19	Y	JR200207_8_IL1	IHC	*I*–*V*
DBC 20	Y	JR200207_10_IL2	IHC	*I*–*V*; sIPSC (L-AP4)
DBC 21	W	JR200311_1_IL1	IHC	-
DBC 22	W	JR200311_2_IL1	reconstruction; EM; IHC	*I*–*V*; sIPSC (L-AP4)
DBC 23	W	JR200311_4_IL2	reconstruction; EM; IHC	*I*–*V*; sIPSC (L-AP4)
DBC 24	W	JR200311_6_IL1	IHC	*I*–*V*
DBC 25	Y	JR200207_11_IL4	EM; IHC	*I*–*V*
DBC 26	Z	JR200219_10_IL2	IHC	*I*–*V*
DBC 27	D	JR181026_3_IL3	IHC	*I*–*V*; eIPSC (pair4)
DBC 28	D	JR181026_3_IL4	IHC	*I*–*V*; eIPSC (pair4)
DBC 29	G	JR190403_1_IL2	IHC	*I*–*V*; eIPSC (pair5)
DBC 30	I	JR190424_8_IL3	IHC	-
DBC 31	X	JR200115_6_IL4	IHC	*I*–*V*; sIPSC (L-AP4)
DBC 32	Y	JR200207_1_IL7	IHC	*I*–*V*
PV-DTC 1	P	JR190927_1_RF1	reconstruction; IHC	*I*–*V*; sIPSC (L-AP4)
PV-DTC 2	R	JR191017_5_RF1	IHC	*I*–*V*; sIPSC (L-AP4)
PV-DTC 3	T	JR191120_1_RF1	EM; IHC	*I*–*V*; sIPSC (L-AP4)
PV-DTC 4	T	JR191120_4_RF4	EM; IHC	*I*–*V*
PV-DTC 5	Q	JR191002_4_RF1	reconstruction; EM; IHC	*I*–*V*; sIPSC (L-AP4)
PV-DTC 6	T	JR191120_2_RF2	IHC	*I*–*V*; sIPSC (L-AP4)
PV-DTC 7	T	JR191120_6_RF1	IHC	*I*–*V*; sIPSC (L-AP4)
PV-DTC 8	E	JR190206_5_IL1	reconstruction; IHC	*I*–*V*
PV-DTC 9	V	JR200110_3_RF2	IHC	*I*–*V*
PV-DTC 10	X	JR200115_3_RF2	IHC	*I*–*V*
PV-DTC 11	x	JR200115_4_RF1	IHC	*I*–*V*
PV-DTC 12	I	JR190424_1_IL3	IHC	sIPSC (L-AP4)
PV-DTC 13	O	JR190904_3_RF1	IHC	-
PV-DTC 14	A	JR171103_12_IL3	reconstruction	-
CTR 1	K	JR190904_4_IL1	-	sIPSC (control)
CTR 2	L	JR190918_3_IL1	-	sIPSC (control)
CTR 3	P	JR190927_2_IL2	-	sIPSC (control)
CTR 4	S	JR191030_3_IL2	-	sIPSC (control)
CTR 5	S	JR191030_6_IL1	-	sIPSC (control)
CTR 6	U	JR191213_2_RF1	-	sIPSC (control)
CTR 7	X	JR200115_7_IL2	-	sIPSC (control)
GBZ 1	D	JR181026_1_IL3	-	sIPSC (gabazine)
GBZ 2	D	JR181026_1_IL4	IHC	sIPSC (gabazine)
GBZ 3	DD	JR181030_1_IL1	IHC	sIPSC (gabazine)
GBZ 4	DD	JR181030_1_IL3	IHC	sIPSC (gabazine)
GBZ 5	EE	JR211110_1_IL2	IHC	sIPSC (gabazine)
GBZ 6	EE	JR211110_4_IL2	IHC	sIPSC (gabazine)
GBZ 7	FF	JR211201_1_IL2	-	sIPSC (gabazine)
GBZ 8	FF	JR211201_3_IL1	IHC	sIPSC (gabazine)
GBZ 9	DD	JR181030_5_IL3	IHC	eIPSC (gabazine)
GBZ 10	DD	JR181030_5_IL4	IHC	eIPSC (gabazine)

CTR—sIPSC control cell; GBZ—cell tested with gabazine; EM—electron microscopy; IHC—immunohistochemistry; I-V—current—voltage relationship; IPSC—inhibitory postsynaptic current (s—spontaneous, e—evoked).

## Results

### Recorded cells and samples

We recorded a total of 622 neurons in vitro in samples from 56 (4 epilepsy, 52 tumor) patients, of which 569 were visualized and fully or partially recovered for anatomical analysis. Of these neurons, 356 (~63%) were smooth dendritic or sparsely spiny interneurons and 213 (~37%) were pyramidal cells, with cell bodies in either layer II or III due to our selection. We further grouped interneurons based on the distribution of their axons and boutons and selected 2 easily distinguishable groups, which had adequate number of cells for comparison. Interneurons with cell body in layer II or upper layer III and a radially oriented axonal plexus, restricted to a narrow (10–20 μm) cortical column descending through layers II–V, were identified as DBCs (*n* = 32, Fig. 1) (Somogyi and Cowey 1981; DeFelipe et al. 2002; DeFelipe 2011). Another group of interneurons had widely distributed axon projecting more than 200 μm in every direction from the soma and large, multipolar dendritic trees. Subsequent analysis showed that these cells express the calcium binding protein parvalbumin (PV); thus, we refer to them as parvalbumin-expressing dendrite-targeting cells (PV-DTCs, *n* = 14, Fig. 4), based on their synaptic targets (see below). We studied the dendritic and axonal distributions, firing patterns, molecular expression, and synaptic connections of these 2 groups of cells in detail to better understand their correspondence to well-defined cell types in other species. Furthermore, we compared the effects of group III mGluR activation on spontaneous GABAergic synaptic inputs in these 2 cell types. A total of 63 neurons were analyzed in samples from 32 patients, including 15 different cortical regions and 14 different diagnoses (Table 2). An additional 3 samples were imported from the University of Szeged, Hungary, for postembedding immunohistochemistry and electron microscopic study of GABAergic synapses (Table 2).

### Axonal and dendritic features and immunohistochemical characterization of DBCs

The distinguishing feature of DBCs is their “horsetail”-like axon (Jones 1975; Somogyi and Cowey 1981; Meyer 1983; Lund and Wu 1997; DeFelipe 2011), formed of at least 3, but often more, interwoven axon branches traveling in the radial direction from the soma in layer II and upper layer III, toward layers V and VI, forming a 10–20 μm wide columnar plexus (Figs. 1a and 6a). We visualized 32 DBCs for anatomical and immunohistochemical analysis. Of these, the current–voltage responses of 29 DBCs were analyzed (Table 3), 3 being excluded because of bad capacitance compensation resulting from changing ACSF levels in the recording chamber.

The axon of DBCs could be traced as deep as layer V (see Fig. 6a). The number of branches in the “horsetails” varied between 3 and 7. Occasionally, short collaterals branched from the “horsetail” at different depths and traveled laterally but remained within 100 μm from the “horsetail” (see Fig. 6a). The majority of DBC boutons were *en-passant*, but all DBCs had a few *en-termineux* boutons on axonal side branches. In addition to the “horsetail,” DBCs also had axon around the cell body and in layer I, where it did not form a columnar structure, but spread 100–500 μm laterally (Fig. 1a). The axon of 22 DBCs entered layer I, with one (DBC 19) exclusively innervating a narrow subpial band. The axon of 9 DBCs spread uniformly across the depth of layer I, whereas another 9 innervated only the deep half. Three DBCs had only a few collaterals entering layer I. The dendrites of 29 DBCs were recovered, of which 24 were smooth, whereas the remainder were sparsely spiny. The dendritic trees were usually small with a median of 4 (range: 2–7) main branches originating from the soma, which traveled mostly toward the pial surface, and less than 100 μm laterally. The distribution of the dendrites was in most cases unipolar toward the pia (*n* = 18), or bipolar in the radial direction (*n* = 9). Two DBCs had uniformly distributed dendritic trees around their soma. All, except DBC 1 had dendrites in layer I.

Previous studies have demonstrated calbindin-immunopositive (CB+) radial axonal columns in human (del Río and DeFelipe 1995, 1997; Ballesteros Yáñez et al. 2005; DeFelipe et al. 2006) and monkey cortex (DeFelipe et al. 1989; DeFelipe et al. 1990; Peters and Sethares 1997), which were predicted to originate from DBCs. We have confirmed the presence of such CB+ axon bundles reminiscent of the “horsetail” axon of DBCs, in the temporal, frontal, and parietal areas of the human neocortex. By labeling individual DBCs with biocytin, we were able to test directly their immunoreactivity for CB and establish the relationship of DBC “horsetails” to axonal bundles revealed by immunoreaction against CB. Furthermore, because interneurons with descending axons were found to be immunoreactive to the calcium binding protein calretinin (CR) and the neuropeptide VIP in rat (Kawaguchi and Kubota 1996), monkey (Lund and Lewis 1993; Condé et al. 1994; Meskenaite 1997; Zaitsev et al. 2005), and human (Varga et al. 2015), we also tested if human DBCs were immunoreactive for these molecules. Other neuropeptides, such as somatostatin (SM) and cholecystokinin (CCK) were also tested. Finally, the calcium binding protein PV was also tested. All DBCs were tested for CB and 24 (75%) were immunopositive in all 3 compartments (axon, soma, and dendrites, *n* = 11), only in their axons (*n* = 8), only in their somata (*n* = 1), or in their somata and axons (*n* = 4). Eleven of these, tested for CR on their axon (*n* = 8), soma (*n* = 1), or both (*n* = 2), were immunonegative. Two DBCs were immunopositive for CR in their axons, and these were immunonegative for CB. Six DBCs were immunonegative for both CR and CB. None of the 14 tested DBCs (8 CB+, 2 CR+, 4 double immunonegative) were immunopositive for VIP in their axons (*n* = 11), somata (*n* = 1), or both (*n* = 2). Similarly, none of the 6 DBCs tested were immunopositive for PV in their axons or CCK in their somata (Fig. 1f). Of the 18 cells tested, only one cell (DBC 18) was immunopositive for SM and this cell was also immunopositive for CB (Fig. 1a, d, f). Interestingly, although there was granular immunoreactivity for SM in the soma of DBC 18, characteristic of peptides found in the Golgi apparatus (Fig. 1a), not all of its boutons were SM-immunoreactive (Fig. 1d). All 16 cells tested were immunopositive for the vesicular GABA transporter in their boutons (VGAT, Fig. 1e and f).

**Fig. 2:**
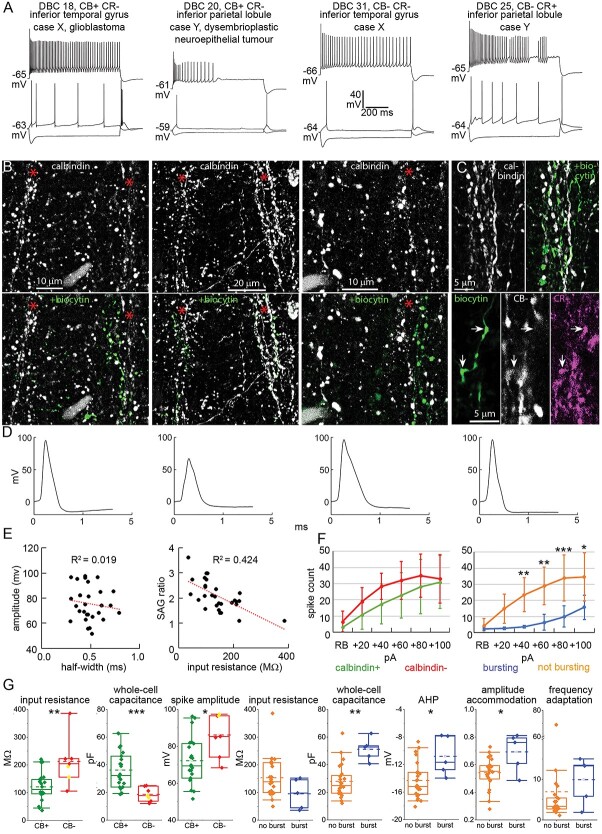
Diversity of DBCs with “horsetail” axons. a) Voltage responses of DBCs to current injections (RB, RB + 100 pA, and holding current—100 pA steps). Immunoreactivity to CB and CR, cortical area and pathology are noted. b) Confocal fluorescent images of immunoreaction to CB (top, white) and overlayed visualization of biocytin (bottom, green), revealing the contribution of individual DBC axons to CB+ axonal columns (asterisk; for DBCs 18, 20, 31, respectively: Z stacks of 32, 25, 7 optical slices of 0.9, 0.9, 0.7 μm thickness, total depth, 13.3, 11.2, 3.0 μm). c) Top: Immunoreaction to CB (white) and overlayed axonal biocytin (green) for the CR+ DBC 25 above (Z stack of 14 optical slices of 0.9 μm thickness, total depth, 5.2 μm); Bottom: CR+ boutons of DBC 25 (arrows) are intermingled with but immunonegative for CB (Z stack of 3 optical slices of 0.9 μm thickness, total depth, 1.6 μm). d) Voltage traces of the first APs evoked at RB current injection for each cell in a. e) Scatter plots of AP amplitude vs. half-width (left, Pearson corr. −0.136, *P* = 0.480); and sag ratio vs. IR (right, Pearson corr. −0.651, *P* = 1.3 * 10^−4^) of all DBCs (*n* = 29, adjusted *R*^2^ values are indicated). f) Firing index (number of spikes elicited by each 800-ms-long current injection step) of CB+ (green) vs. CB- (red) DBCs (left, 2-way repeated measures ANOVA, within subjects effect of current step: *F* = 50.712, *P* = 1.1 * 10^−7^; within subjects effect of interaction: *F* = 1.353, *P* = 0.246; between subjects effect of immunoreactivity: *F* = 2.164, *P* = 0.153) and of bursting (blue) vs. not bursting (orange) DBCs (right, 2-way repeated measures ANOVA, within subjects effect of current step: *F* = 28.365, *P* = 1.2 * 10^−5^; within subjects effect of interaction: *F* = 7.201, *P* = 5.3*10^−6^; between subjects effect of bursting: *F* = 13.840, *P* = 9.2 * 10^−4^, Tukey pairwise comparisons: bursting vs. not bursting within each current step: ^*^P < 0.05, ^**^P < 0.01, ^***^P < 0.001; whiskers represent SD). g) Comparison of passive and active membrane properties between CB+, (green, *n* = 22) and CB− (red, *n* = 7) DBCs (CR+ cells, yellow), and between bursting (blue, *n* = 5) and not bursting (regular firing, orange, *n* = 24) DBCs. Boxes represent median and IQR, whiskers represent 5 and 95 percentiles, mean is indicated by dashed line. For all CB+ vs. CB−, and the AHP and AP amplitude accommodation comparisons student’s *t*-test was used, for the remainder Mann–Whitney U-test, ^*^P < 0.05, ^**^P < 0.01, ^***^P < 0.001.

We found CB+ DBCs in the temporal (*n* = 13), frontal (*n* = 4), parietal (*n* = 4), and parieto-occippital (*n* = 3) cortices. Calbindin-immunonegative DBCs were found in the temporal (*n* = 4) and parietal (*n* = 4) cortices. One CR+ DBC was found in the temporal, whereas the other in the parietal cortex. The only SM immunopositive DBC was in the temporal cortex. The “horsetail” axons of the recorded and biocytin-labeled CB+ DBCs always aligned with the CB+ axonal bundles, where they intermingled with CB+ axons from unknown neurons (Fig. 2b, 1st and 2nd columns). Furthermore, the axons of CR+ and CB-/CR- double immunonegative DBCs also aligned with and traveled within CB+ bundles (Fig. 2b, 3rd column and Fig. 2c). While most DBCs’ axon formed one “horsetail,” DBCs 1 and 4 formed 2, and DBC 20 formed 3 separate “horsetails”, each aligned with a different CB+ bundle (Fig. 2b, 2nd column). However, cells with one main “horsetail” would also give off collateral branches leaving the main CB+ bundle and entering a neighboring one, forming boutons also between bundles. Finally, in one case, where 2 DBCs (DBCs 27 and 28) were labeled close to each other, their axons intermingled to form a single “horsetail.” These results show that the cortical columns determined by the CB+ bundles are complex structures including the “horsetail” axons of multiple CB+ as well as CR+ DBCs, among other cells.

### Intrinsic membrane properties of DBCs

**Fig. 3:**
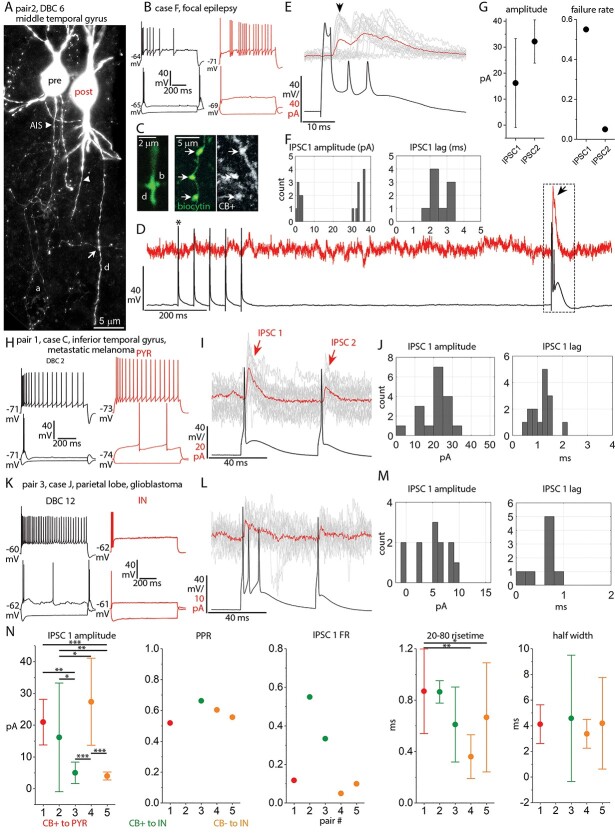
Synaptic currents evoked by DBCs. a) Fluorescent image of 2 simultaneously recorded synaptically connected interneurons (pair 2). The presynaptic DBC (pre) has a “horsetail” axon (a) and a collateral forms a bouton in close apposition to the dendrite (d, arrow) of the postsynaptic interneuron (post). AISs, arrowheads. (Z stack of 16 optical slices of 4.3-μm thickness, total depth, 42.8 μm). b) Voltage responses of the pre- (black) and postsynaptic (red) cells in a to current injections of RB, RB +100 and holding current-100 pA. c) **Left:** Fluorescent image of one contact site (arrow in a) showing the putative presynaptic bouton (b) in close juxtaposition with the postsynaptic dendrite (d, single optical slice of 0.7-μm thickness). **Right:** Boutons (arrows) of the presynaptic DBC (green) are CB+ (gray) (single optical slice of 0.9-μm thickness). d) Example of the current response in the postsynaptic cell (red, single sweep) voltage clamped at −47 mV to a train of single APs (asterisk) 50 ms apart and an AP burst (boxed area) evoked in the presynaptic DBC (black) in current clamp. There is an IPSC response to the burst (arrow) but not to the single APs (failures). e) IPSC responses in the postsynaptic cell (single responses in gray, arrow 1st response, average of 20 responses in red) to the AP burst in the presynaptic DBC (black, single sweep). f) Distributions of the peak amplitude (*n* = 20, including failures) and response lag (*n* = 9, excluding failures) of the IPSC after the first AP in the burst (bin width: 1 pA for amplitude and 0.5 ms for lag). g) Mean response amplitude including failures (left) and failure rate (right) of the IPSCs after the first and second APs in the burst (*n* = 20 for each of IPSC1 and 2, whiskers represent SD). h) Voltage responses of the presynaptic DBC 2 (black) and postsynaptic pyramidal cell (red) of Pair 1 to current injections of RB, RB +100 and holding current −100 pA. i) Current responses in the postsynaptic cell of pair 1 (single responses in gray, average of 17 responses in red) to a pair of APs evoked in the presynaptic DBC 2 (black) 50 ms apart. j) Distribution of peak amplitude (*n* = 17, including failures) and response lag (*n* = 15, excluding failures) of IPSC 1 (bin width: 5 pA for amplitude and 0.2 ms for lag). k–m) Same as h–j for pair 3 (bin width: 1 pA for amplitude and 0.2 ms for lag; *n* = 12 including, *n* = 8 excluding failures). The presynaptic DBC fires AP bursts to rheobase current injection and the postsynaptic cell is an interneuron (IN). n) Mean amplitude (including failures) of IPSC 1 (*n* = 17, 20, 12, 20, and 20 responses for neuron pairs 1–5, respectively; 1-way ANOVA, *F* = 16.22, *P* = 4.7 * 10^−8^, Tukey post-hoc test; *P* < 0.05 for pairs 4 vs. 2 and 3 vs. 2; *P* < 0.01 for pairs 5 vs. 2 and 3 vs. 1; *P* < 0.001 for pairs 4 vs. 3, 4 vs. 5 and 5 vs. 1), paired-pulse ratio (PPR, average response2/response1 mean amplitude), failure rate, 20–80% rise-time (1-way ANOVA, *F* = 7.37, *P* = 3 * 10^−4^, Tukey post-hoc test, *P* < 0.05 for pair 5 vs. 1; *P* < 0.001 for pair 4 vs. 1) and half-width (1-way ANOVA, *F* = 0.47, *P* = 0.707) of IPSC 1 for 5 DBC synapses onto different types of postsynaptic neuron. Whiskers represent SD, plots are color coded according to immunoreactivity of the presynaptic DBC for CB and the postsynaptic neuron class (PYR: pyramidal cell; IN: interneuron).

We have found that DBCs express firing phenotypes that distinguish them from PV-DTCs and other cell interneuron types in the neocortex. All DBCs, except DBCs 28 and 29, responded with a prominent voltage sag (Figs. 1b, 2a , 3b, h, and k, 5a; sag ratio: 2.07 ± 0.58, *n* = 29) and 19 of 32 DBCs also fired rebound spikes after continuous hyperpolarization (Figs. 2a, 3b and k, and 5A). Five of 32 DBCs showed burst firing either at rheobase current injection and/or as rebound after continuous hyperpolarization (Fig. 1b), and all 5 were CB+. The recorded CB− DBCs did not show burst firing. When continuously depolarized by rheobase +100-pA current injection, 14 DBCs fired a regular train of APs (Fig. 2a, DBCs 18 and 31), 5 showed depolarisation block (Fig. 2a, DBC 20), and 7 fired either irregular or stuttering AP trains (Fig. 2a, DBC 25). All DBCs showed strong spike amplitude accommodation (average 0.57 ± 0.12) and spike frequency adaptation (median: 3.43, IQR: 3.52). At rheobase +100-pA current injection, DBCs fired at an average firing rate of 38.1 ± 19.7 Hz, but their coefficient of variation of the inter-spike-intervals (ISI-CV), a measure of the regularity of the spike train, varied between 0.09 for the most regularly firing cell (DBC 24) and 2.06 for DBC 11, with a stuttering firing pattern (average: 0.47 ± 0.39).

The AP kinetics of DBCs were variable (Fig. 2d). Overshooting AP amplitude varied between 52 and 97 mV, the average being 75 ± 2.5 mV (*n* = 29). The resting membrane potential was −56.8 ± 4.3 mV. The AP half-width varied between 0.29 and 0.79 ms, the average being 0.48 ± 0.02 ms, as measured on the first elicited AP at rheobase current injection. There was no correlation between AP amplitude and half-width (Fig. 2e, left, Pearson corr. −0.136, *P* = 0.480). IR and sag ratio also varied across DBCs and were inversely correlated (Fig. 2e, right, Pearson corr. −0.651, *P* = 1.3 * 10^−4^).

Next, we compared the firing patterns, the passive and active membrane properties of CB+ (*n* = 22) vs. CB− DBCs (*n* = 7), and bursting (*n* = 5) vs. regular firing (not bursting) DBCs (*n* = 24). The firing index plots (number of APs per current step) indicated no difference in input gain between CB+ and CB− DBCs (Fig. 2f, left, 2-way repeated measures ANOVA, within subjects effect of current step: *F* = 50.712, *P* = 1.1 ^*^ 10^−7^; within subjects effect of interaction: *F* = 1.353, *P* = 0.246; between subjects effect of immunoreactivity: *F* = 2.164, *P* = 0.153). Furthermore, those CB+ DBCs, which showed a bursting phenotype, fired significantly fewer APs than regular firing DBCs, which include both CB+ and CB− DBCs, at every current injection step above rheobase +20 pA (Fig. 2f, right, 2-way repeated measures ANOVA, within subjects effect of current step: *F* = 28.365, *P* = 1.2 * 10^−5^; within subjects effect of interaction: *F* = 7.201, *P* = 5.3*10^−6^; between subjects effect of bursting: *F* = 13.840, *P* = 9.2 ^*^ 10^−4^, Tukey pairwise comparisons: bursting vs. not bursting within each current step: ^*^P < 0.05, ^**^P < 0.01, ^***^P < 0.001). Indeed, bursting DBCs often ceased firing after the initial burst even at large depolarizing current injections. The IR of CB+ DBCs was lower than that of CB− DBCs (Fig. 2g, 121.1 ± 50.7 MΩ for CB+ DBCs vs. 212.9 ± 86.8 MΩ for CB− DBCs, student’s *t*-test *t*_(27)_ = −3.49, *P* = 0.002). Conversely, whole-cell capacitance was higher in CB+ DBCs than in CB− ones (36.2 ± 13.8 pF for CB+ DBCs vs. 18.6 ± 5.1 pF for CB− DBCs, Welch’s *t*-test *t*_(26.07)_ = 4.98, *P* = 3.5 * 10^−5^), indicating that CB+ DBCs are larger cells with bigger surface area. Finally, AP amplitude was larger in CB− DBCs than in CB+ ones (72.2 ± 12.5 mV for CB+ DBCs vs. 86.2 ± 11.6 mV for CB− DBCs, student’s *t*-test *t*_(27)_ = −2.61, *P* = 0.014). The sag ratio, AP threshold, AP half-width, AHP, and the ISI CV were not different between CB+ and CB− DBCs.

Bursting DBCs had similar IR to regular firing (not bursting) DBCs (Fig. 2g, median: 138.5, IQR: 110.2 MΩ for not bursting vs. median: 97.8, IQR: 108.6 MΩ for bursting, Mann–Whitney U test Z_(88)_ = 1.59, *P* = 0.112), whereas the whole-cell capacitance was higher in bursting DBCs than in regular firing ones (median: 24.8, IQR: 13.4 pF for not bursting vs. median: 52.8, IQR: 14.7 pF for bursting, Mann–Whitney U test Z_(8)_ = −2.97, *P* = 0.003). The amplitude of the AHP was larger in nonbursting DBCs than in bursting ones (−14.3 ± 2.6 mV for not bursting vs. −10.8 ± 2.8 mV for bursting, student’s *t*-test *t*_(27)_ = −2.71, *P* = 0.012). Finally, not bursting DBCs showed stronger AP amplitude accommodation than bursting DBCs (0.54 ± 0.11 for not bursting vs. 0.69 ± 0.14 for bursting, student’s *t*-test *t*_(27)_ = −2.72, *P* = 0.011), whereas AP frequency adaptation was not different (Fig. 2g; median: 3.36, IQR: 2.78 for not bursting vs. median: 13.3, IQR: 10.39 for bursting, Mann–Whitney U test Z_(30)_ = −1.70, *P* = 0.089).

### *Spontaneous and evoked IPSCs are mediated by GABA*_*A*_  *receptors*

We tested if GABAergic synaptic transmission to different types of interneuron in the human neocortex is differentially regulated by group III mGluRs. Specifically, we tested whether activation of group III mGluRs by the orthosteric agonist *L-2-amino-4-phosphonobutyric acid* (L-AP4) changes the frequency and/or amplitude of GABA_A_ receptor-mediated sIPSCs, as has been shown in rodents (Mitchell and Silver 2000; Giustizieri et al. 2005; Cuomo et al. 2009). We refer to outward currents as sIPSCs, because under our experimental conditions in 6 interneurons (4 visualized) and 2 pyramidal cells, the GABA_A_ receptor antagonist gabazine (16 μM) completely abolished outward currents, without changing inward currents (Supplementary Fig. 1a and see Materials and methods). Gabazine also abolished evoked outward currents in a monosynaptic connection between 2 interneurons, indicating that these were mediated by GABA_A_ receptors. Therefore, we refer to these currents as eIPSCs (Supplementary Fig. 1b). It is important to note that APs and fast glutamatergic synaptic transmission were not blocked in these experiments.

### Unitary synaptic currents evoked by DBCs

The target cell types and subcellular target domains of DBC efferent synapses and their postsynaptic effects are not known in humans. Following previous work in other species (Tamás et al. 1997a; Jiang et al. 2015), we tested unitary synaptic interactions between pairs of neurons consisting of one DBC and one other GABAergic interneuron or a pyramidal cell within a 50-μm distance from the DBC soma. We tentatively identified DBCs during the recordings based on their characteristic firing. We succeeded in testing 12 unitary synaptic connections from DBCs to interneurons and 4 connections to pyramidal cells and detected postsynaptic responses in 3 interneurons (DBC-to-IN) and 1 pyramidal cell (DBC-to-PYR), a 25% connectivity ratio (Fig. 3). We also found 2 unitary synaptic connections from interneurons to DBCs out of 9 tested.

The identity of presynaptic DBCs (*n* = 5) was confirmed by light microscopy based on their “horsetail” axon (Fig. 3a). Three of them (DBCs 2, 6, and 12) were immunopositive for CB (Fig. 3c); the other 2 (DBCs 28 and 29) were CB-/CR- double immunonegative (see Fig. 1f). The cell bodies of the recorded cell pairs were located in layers II or III. One of the postsynaptic interneurons (pair 4) was also identified as a CB-/CR- double immunonegative DBC (DBC 27, see Fig. 1f); there was no detectable connection from DBC 27 to DBC 28. We were not able to determine the cell type of the other 3 postsynaptic interneurons. One of them (pair 2) had a bipolar dendritic tree originating from 2 main trunks at the superficial and deep poles of the soma (Fig. 3a), and its axon had loose branches descending toward deeper layers. Its firing pattern was stuttering and showed spike amplitude accommodation; it had no sag potential (Fig. 3b). In rodents, similar neurons are thought to express VIP. However, this postsynaptic human interneuron was double immunonegative for VIP as well as for CB. In pair 3, the postsynaptic cell had a firing pattern resembling that of multipolar basket cells, such as high frequency regular firing and depolarization block (Fig. 3k), had a small sag potential and a multipolar dendritic tree. In contrast, the postsynaptic interneuron in pair 5 had a slow irregular firing pattern, no sag potential and dendrites oriented radially. This cell was immunonegative for CR, VIP, and CB. The axons of 2 postsynaptic interneurons (pairs 3 and 5) were not recovered. In pair 1, the postsynaptic pyramidal cell was identified based on its densely spiny dendrites.

In order to test for unitary synaptic connections, we evoked pairs or trains (max. 5) of APs 50 ms apart in one cell held in current clamp between −70 and −75 mV by brief (1–5 ms) current injections of 100–500 pA while recording the other neuron in voltage-clamp mode at just below firing threshold potentials (−45 to −50 mV). The peak amplitude of the evoked inhibitory postsynaptic currents (eIPSCs), the paired-pulse ratio (PPR, amplitude of IPSC2/amplitude IPSC1), and the failure rate of the first eIPSC were evaluated, as well as the 20–80% risetime and the half-width of the first eIPSC. In pair 2, single APs evoked 50 ms apart in the presynaptic DBC did not elicit IPSCs in the postsynaptic cell; only evoking a presynaptic AP burst resulted in detectable bursts of postsynaptic IPSCs (Fig. 3d and e). In this case, the PPR was not available. In all other pairs, single APs elicited IPSCs in the postsynaptic cells. However, in pair 3, although single APs could elicit detectable IPSCs, the first presynaptic stimulus was always a burst of 2–3 APs, even at the lowest current injection (Fig. 3l).

We found large variability in the response parameters of the 5 synaptic connections made by DBCs onto interneurons and pyramidal cells. The mean amplitude of the first eIPSC in the DBC-to-PYR connection (pair 1) was 21 ± 1.7 pA (*n* = 17, including failures), with a failure rate of 0.12 and a PPR of 0.52. In the DBC-to-IN connections, the amplitude was 16.2 ± 3.8 pA (*n* = 20, including failures) in pair 2, and it was significantly higher in pair 4 (27.4 ± 3.1 pA, *n* = 20, including failures) and was significantly lower in pairs 3 (5 ± 0.9 pA, *n* = 12, including failures) and 5 (4 ± 0.3 pA, *n* = 20, including failures; 1-way ANOVA, *F* = 16.22, *P* = 4.7 * 10^−8^, Tukey post-hoc test; *P* < 0.05 for pairs 4 vs. 2 and 3 vs. 2; *P* < 0.01 for pairs 5 vs. 2 and 3 vs. 1; *P* < 0.001 for pairs 4 vs. 3, 4 vs. 5, and 5 vs. 1; Fig. 3n, IPSC 1 amplitude). Conversely, IPSC1 failure rates were low in pairs 4 and 5 (0.05 and 0.1, respectively) and higher in pairs 2 and 3 (0.55 and 0.33, respectively; Fig. 3n, IPSC1 FR). All pairs, except pair 2 showed paired-pulse depression of eIPSC amplitude, the PPR being 0.66, 0.6, and 0.56 for pairs 3, 4, and 5, respectively (Fig. 3n, PPR), indicating a decrease of release probability from AP1 to AP2. In contrast, in pair 2, where the presynaptic input was a burst of APs, the delay between the first and second APs within the burst varied around 10 ms. Here, we measured paired-pulse facilitation, from 16.2 ± 3.8 pA (*n* = 20, including failures) of IPSC1 to 32.2 ± 1.9 pA (*n* = 20, including failures) of IPSC2 (Fig. 3e and g). This paired-pulse facilitation was accompanied by a decrease in failure rate of IPSC2 compared with IPSC1 from 0.55 to 0.5 (Fig. 3g). The increased release probability at AP2 is likely explained by the short (~10 ms) delay between the first 2 APs in the burst and accumulation of intracellular calcium.

We also analyzed the response kinetics of these connections. The largest response delay (from AP peak to IPSC onset) was in pair 2 (2.5 ± 0.2 ms, *n* = 9 IPSCs, excluding failures; Fig. 3f), and the shortest in pair 3 (0.6 ± 0.1 ms, *n* = 8 IPSCs, excluding failures; Fig. 3m); IPSC1 delay in pairs 1, 4, and 5 was 1.2 ± 0.1 (*n* = 15), 1.6 ± 0.05 (*n* = 19), and 1.8 ± 0.1 ms (*n* = 18), respectively (1-way ANOVA, *F* = 34.75, *P* = 2.1 * 10^−15^, Tukey post-hoc test, *P* < 0.05 for all comparisons). The mean risetime (20–80%) was the shortest in pair 4 with 0.36 ± 0.039 ms (*n* = 19). Pairs 3 and 5 had a risetime of 0.61 ± 0.1 (*n* = 8) and 0.67 ± 0.1 ms (*n* = 18 IPSCs, excluding failures), respectively. In pair 1, the risetime was 0.87 ± 0.058 ms (*n* = 15 IPSCS, no failures) (1-way ANOVA, *F* = 7.37, *P* = 3 * 10^−4^, Tukey post-hoc test, *P* < 0.05 for pair 5 vs. 1; *P* < 0.001 for pair 4 vs. 1; Fig. 3n, 20–80 risetime). Finally, we found no difference in half width between the 4 connections (pair 2 excluded due to burst) (1-way ANOVA, *F* = 0.47, *P* = 0.707; Fig. 3n, half-width).

In summary, the most reliable connection was made between DBC 28 and DBC 27 (pair 4), and this synapse also showed the fastest kinetics. Connections made by CB+ DBCs to interneurons were less reliable than other connections. Finally, the connection by the CB+ DBC onto the pyramidal cell was also reliable but showed slower kinetics than the connection in pair 4.

### Axonal and dendritic features and molecular characterization of PV-DTCs

**Fig. 4:**
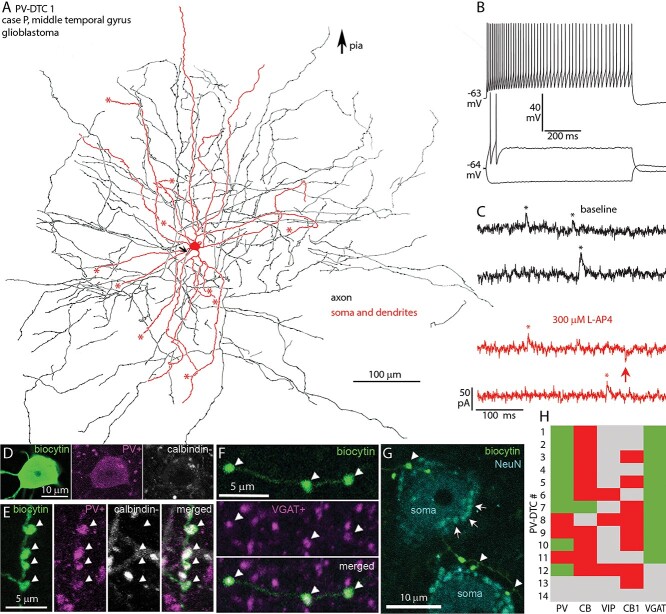
In vitro recorded and biocytin labeled PV-DTC in the human temporal neocortex. a) Partial 2D reconstruction (2 sections of ~60-μm thickness) of the cell with cell body in layer III; the axon did not enter layer I. The AIS (arrow), cut ends of dendrites (asterisks), and direction of the pial surface are indicated. b) Voltage responses of the cell in A to current injections of RB, RB +150, and holding current −150 pA. Firing is a regular train of APs with spike frequency adaptation and small spike amplitude accommodation. There is a minimal voltage sag and no rebound spikes in response to hyperpolarising current injection. c) Example current traces recorded in voltage-clamp mode at −50 mV clamping potential, showing sIPSCs (asterisks, upward deflections) and sEPSCs (arrow, downward deflections) in baseline condition (black) and after bath application of 300 μM L-AP4 (red). d) The cell body is immunopositive for PV (magenta) and immunonegative for CB (white) in the cytoplasm (Z stack of 9 optical slices of 0.9-μm thickness, total depth, 4.7 μm). e) Boutons of the cell in A are immunopositive for PV (magenta, arrowheads) and immunonegative for CB (white) (Z stack of 13 optical slices of 0.9-μm thickness, total depth, 7.9 μm). f) Boutons (arrowheads) of the cell in a are immunopositive for VGAT (magenta) (single optical slice of 0.7-μm thickness). g) Boutons (arrowheads) of the cell in a (biocytin, green) are in close proximity with NeuN (cyan) immunopositive cell bodies (soma), which also contain autofluorescent lipofuscin granules (e.g. arrows). (Z stack of 4 optical slices of 0.7-μm thickness, total depth, 2.4 μm). h) Immunoreactivities in all recovered and tested neurons with multipolar dendrites: green, immunopositive; red, immunonegative; gray, not tested/inconclusive.

We have fully recovered 14 (12 cells in temporal and 2 cells in the frontal cortex) cells with large, multipolar, smooth dendritic trees, and extensive axon spreading uniformly in all directions from the soma (Fig. 4a). The cell bodies of these cells were located in layer III at an average distance of ~1000 ± 430 μm (*n* = 3) from the pia. The 6–8 main dendrites traveled radially on average to ~375 ± 37 μm (*n* = 4) from the soma and had a relatively straight course (Fig. 4a). The axons of these cells were even more extensive and traveled to ~500 ± 97 μm on average uniformly in every direction (Fig. 4a). Importantly, the axons did not project to layer I and formed mostly *en-passant* and only few *en-termineux* boutons, which occasionally contacted neuronal somata (Fig. 4g).

Neurons with similar dendritic and axonal distributions have been described previously in humans and other species. One such interneuron type is the *large basket cell*. Basket cells innervate the somata and the proximal dendrites of pyramidal neurons, as well as dendritic spines (Somogyi et al. 1983; Kisvarday et al. 1987; Kisvárday et al. 1990; Kisvárday et al. 1993). However, cells with similar dendritic trees and axonal distribution have also been found innervating mostly dendrites, including spines and no cell bodies (Tamás et al. 1997a). Furthermore, basket cells have been demonstrated previously to be of at least 2 types in rodents, which can be differentiated based on immunoreactivity to PV or CCK (Freund et al. 1983, 1986; Kawaguchi and Kubota 1998; Somogyi et al. 2004; Armstrong and Soltész 2012). Those that are immunopositive for CCK have been found to be also immunopositive for the cannabinoid type 1 receptor (CB1R) along the axonal membrane in the hippocampus (Katona et al. 1999). Thus, we tested our multipolar cells for immunoreactivity to PV and CB1. We also tested them for CB, as this was previously found to be co-expressed with PV or CCK in interneurons (Gulyás et al. 1991; Kubota et al. 1994), and 9 out of 12 tested multipolar cells were immunopositive for PV as shown either in their cell body (*n* = 2) or boutons (*n* = 10; Fig. 4d, e, and h), whereas none of the 5 PV+, 3 PV−, and 1 PV-untested cells were immunopositive for CB1 along their axons (Fig. 4h), and 8 of the 9 PV+ multipolar cells were immunonegative for CB in their boutons (*n* = 5) or soma (*n* = 3, Fig. 4e); one was immunopositive in the soma. Two PV− multipolar cells tested were also immunonegative for CB in their boutons. The boutons of 2 PV+ and one PV− multipolar cell were tested for VIP and were immunonegative. The boutons of all 11 multipolar cells tested were immunopositive for VGAT (Fig. 4f and h).

The expression of PV by the majority of the multipolar cells and their axonal and dendritic structures are characteristic of neocortical *large basket cells*, and *dendrite-targeting cells* described in other species (Hartwich et al. 2009). However, because we found the majority of our multipolar cell synapses innervating dendrites and only a few synapses targeting somata (see later), instead of naming them basket cells, which might be misleading, we refer to them as PV-DTCs.

### Intrinsic membrane properties of PV-DTCs in comparison with DBCs

**Fig. 5:**
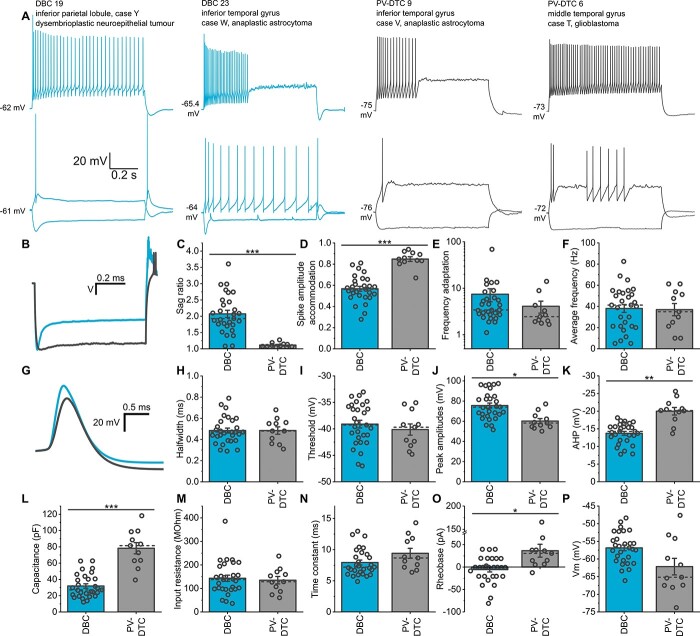
Comparison of membrane properties of DBCs and PV-DTCs. a) Example traces from 2 DBCs (blue) and 2 PV-DTCs (gray) showing voltage responses to current injections of rheobase, holding current −100 pA and rheobase +100 pA. b) Average waveforms of the voltage responses of the DBCs (blue, *n* = 29) and PV-DTCs (gray, *n* = 11) to holding current −100 pA current injection. The average traces were normalized such that the initial baseline values and the lowest points on the voltage responses are equal. c) Ratios of the sags in the voltage responses to stimulation with holding current −100 pA, calculated as the peak voltage deflection divided by the amplitude of the steady state voltage response (Welch’s *t*-test, *t*_(29.9)_ = 8.55, *P* = 1.6 × 10^−9^). d) Spike amplitude accommodation ratios, calculated by dividing the amplitude of the last AP by the amplitude of the first generated in response to rheobase +100 pA current injection (Student’s *t*-test, *t*_(38)_ = −6.98, *P* = 2.6 × 10^−8^). e) Firing frequency adaptation ratios calculated by dividing the last inter-spike interval with the first one of the response to rheobase +100 pA current injection (Kolmogorov–Smirnov test, *D* = 0.429, *Z* = 1.21, *P* = 0.074). f) Average frequencies of APs generated in an 800 ms response to rheobase +100 pA current injection (Student’s *t*-test, *t*_(38)_ = 0.16, *P* = 0.87). g) Average waveforms of the first APs generated at rheobase current injection for each cell. h–j) Halfwidths (h, Student’s *t*-test, *t*_(38)_ = −0.008, *P* = 0.99), thresholds (i, Student’s *t*-test, *t*_(38)_ = 0.76, *P* = 0.45), and amplitudes (j, Student’s *t*-test, *t*_(38)_ = 3.5, *P* = 0.001), of the first AP generated in response to rheobase current injection for each cell. k**)** After hyperpolarization amplitudes of the first AP for each cell at rheobase current injection, calculated relative to the threshold of that AP (Student’s *t*-test, *t*_(38)_ = 5.71, *P* = 1.4 × 10^−6^). l) Whole cell capacitances, calculated from the voltage amplitude and the time constant associated with the slowest component of a double exponential fit to the initial voltage deflections of the responses to holding current −60 pA current injection (Student’s *t*-test, *t*_(38)_ = −7.69, *P* = 2.9 × 10^−9^). m) Comparison of IRs (Student’s *t*-test, *t*_(38)_ = 0.34, *P* = 0.733). n) Membrane time constants, measured as the slowest component of the double exponential fit described in l (Student’s *t*-test, *t*_(38)_ = −1.82, *P* = 0.077). o) The amount of current required to elicit at least one AP within an 800-ms period of stimulation for each cell (Welch’s *t*-test, *t*_(12.89)_ = −2.74, *P* = 0.017). Note that 20-pA current injection steps were used, and the rheobase was taken as the holding current of the first step to elicit an AP. p) The membrane voltage of each cell recorded in response to a 0-pA current injection or estimated based on the cell’s current–voltage relationship if no 0-pA step was recorded (Welch’s *t*-test, *t*_(12.49)_ = 2.13, *P* = 0.053).

Based on recording quality, the current–voltage responses of 11 of the PV-DTCs were analyzed (Table 3). Several active and passive membrane properties differed between PV-DTCs and DBCs; PV-DTCs showed a smaller amplitude voltage sag in response to hyperpolarizing current injection than DBCs and lacked rebound spikes after the hyperpolarizing steps (Figs. 4b and 5a–c, sag ratio: PV-DTCs 1.11 ± 0.06 vs. DBCs 2.07 ± 0.58, Welch’s *t*-test *t*_(29.9)_ = 8.55, *P* = 1.6 × 10^−9^). Only the PV+/CB+ double immunopositive PV-DTC 7 fired rebound spikes. During continuous depolarisation, PV−DTCs fired a regular train of APs with less spike amplitude accommodation than DBCs (Fig. 5d; PV-DTCs 0.85 ± 0.07 vs. DBCs 0.57 ± 0.12, Student’s *t*-test *t*_(38)_ = −6.98, *P* = 2.6 × 10^−8^); spike frequency adaptation was similar in the 2 cell types (Fig. 5e; PV-DTCs median: 2.45, IQR: 1.83, DBCs median: 3.43, IQR: 3.52, Kolmogorov–Smirnov test *D* = 0.429, *Z* = 1.21, *P* = 0.074). Two PV-DTCs developed depolarization block at RB + 100 pA current injection. In contrast to DBCs, only 1 cell, PV-DTC 2, responded with a burst of 4 APs, all other PV-DTCs fired 1 or 2 APs to RB current (e.g. Fig. 4b, PV-DTC 1 and Fig. 5a, PV-DTC 9). The mean firing rate of PV-DTCs at RB +100 pA current injection was similar to that of DBCs (PV-DTCs 36.9 ± 18.3 Hz vs. DBCs 38.1 ± 19.7 Hz, Student’s *t*-test *t*_(38)_ = 0.161, *P* = 0.87), but the regularity of the AP trains was different, as the coefficient of variation of the inter-spike intervals (ISI CV) was smaller in PV-DTCs than DBCs (PV-DTCs median: 0.2, IQR: 0.08 vs. DBCs median: 0.33, IQR: 0.33, Kolmogorov–Smirnov test *D* = 0.508, *Z* = 1.434, *P* = 0.021).

Parvalbumin-expressing basket cells in the cortex of other species are also referred to as fast-spiking basket cells, because of their short duration APs and high frequency firing in vivo and in vitro (McCormick et al. 1985; Baranyi et al. 1993; Kawaguchi 1995; Erisir et al. 1999). In our sample, PV-DTCs had a mean AP half-width of 0.48 ± 0.12 ms, similar to that of DBCs (Fig. 5g and h; 0.48 ± 0.11 ms, Student’s *t*-test *t*_(38)_ = −0.008, *P* = 0.99). Under our conditions, the AP threshold of PV-DTCs was not different from that of DBCs (Fig. 5i; PV-DTCs −40.1 ± 3.4 mV vs. DBCs −39.1 ± 3.8 mV, Student’s *t*-test *t*_(38)_ = 0.76, *P* = 0.45), but on average PV-DTCs had smaller AP amplitude than DBCs (Fig. 5j; PV-DTCs 60.2 ± 7.9 vs. DBCs 75.6 ± 13.3 mV, Student’s *t*-test *t*_(38)_ = 3.5, *P* = 0.001) and larger AHP amplitude (Fig. 5k; PV-DTCs 20 ± 3.4 mV vs. DBCs 13.7 ± 2.9 mV, Student’s *t*-test *t*_(38)_ = 5.71, *P* = 1.4 × 10^−6^).

PV-DTCs had larger whole-cell capacitance than DBCs (Fig. 5l; PV-DTCs 78.3 ± 21.8 pF vs. DBCs 31.9 ± 14.1 pF, Student’s *t*-test *t*_(38)_ = −7.69, *P* = 2.9 × 10^−9^), reflecting the larger cell bodies and longer dendrites of PV-DTCs compared with DBCs. The IR and membrane time constant of PV-DTCs was not different from those of DBCs (Fig. 5m, IR; PV-DTCs 135 ± 50 MΩ vs. DBCs 143 ± 70 MΩ, Student’s *t*-test *t*_(38)_ = 0.343, *P* = 0.733; Fig. 5n, tau; PV-DTCs 9.4 ± 2.5 ms vs. DBCs 7.9 ± 2.1 ms, Student’s *t*-test *t*_(38)_ = −1.82, *P* = 0.077). Furthermore, PV-DTCs had larger rheobase than DBCs (Fig. 5o; PV-DTCs 36.5 ± 45.5 pA vs. DBCs −5.5 ± 28.4 pA, Welch’s *t*-test *t*_(12.89)_ = −2.74, *P* = 0.017), whereas their resting membrane potentials were similar (Fig. 5p; PV-DTCs −62.2 ± 7.4 mV vs. DBCs 56.9 ± 4.3 mV, Welch’s *t*-test *t*_(12.49)_ = 2.133, *P* = 0.053).

### Synaptic targets of DBCs and PV-DTCs

In nonhuman species, DBCs innervate both pyramidal cells and other interneurons (Somogyi and Cowey 1981; DeFelipe et al. 1989; DeFelipe and Jones 1992; Tamás et al. 1997a), in agreement with our paired IPSC recordings in humans. The connectivity rate of 25% for interneurons in the vicinity of DBCs may be an underestimate due to dendritic filtering of small IPSCs. We could not record a representative sample of DBC-to-PYR connections to estimate the connectivity rate. Furthermore, because our paired recordings were restricted to the vicinity of the DBC soma in layers II–III, we do not know if these interactions were mediated by the “horsetail” of DBCs or the more loosely distributed axon collaterals in the vicinity of the DBC somata.

**Fig. 6:**
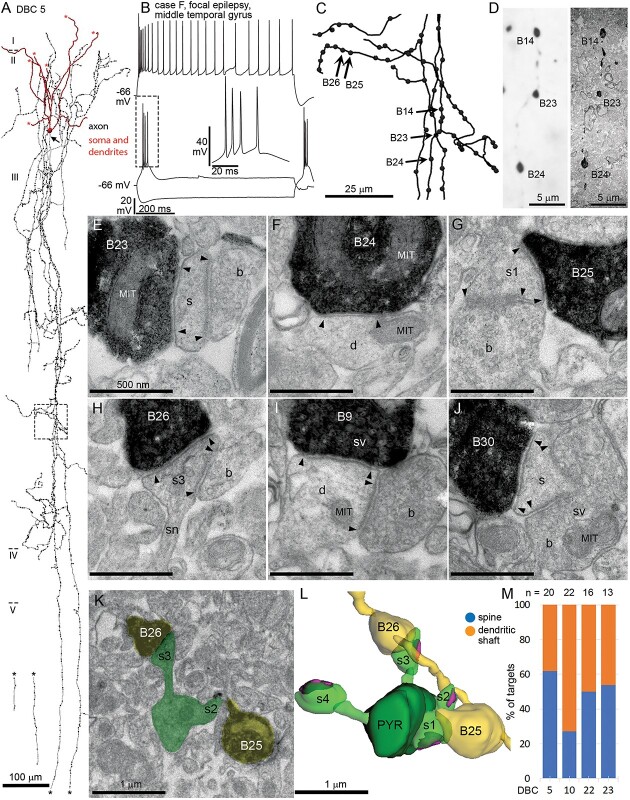
Synaptic targets of DBCs in layer III of the human neocortex. a) Partial 3D reconstruction (3 sections of ~60-μm thickness) of DBC5 with dendrites entering layer I, and a “horsetail” axon (arrow, initial segment) descending to layer V (asterisks indicate cut ends of axon and dendrites); note short lateral collaterals in layer III. b) Voltage responses of the cell to current injections (RB, RB+100 and holding current −100 pA steps) in whole-cell patch-clamp; inset showing the burst in boxed area. c) Enlarged view of the boxed area in a, included in serial electron microscopic analysis; numbered boutons are shown in e–h, k–l. d) **Left:** Light micrograph of a descending axon collateral with 3 boutons visualized by HRP reaction product (single optical section within a ~60-μm-thick osmium-treated section); **Right:** low power electron micrograph of the same boutons in a 50-nm-thick resin section. e–j) High power electron micrographs of the boutons of the cell in a forming type-2 synapses with their targets. The edges of synapses are indicated by black arrowheads. Note the smooth apposition of the pre- and postsynaptic membranes and synaptic vesicles (sv) at the presynaptic active zones. Boutons 23, 25, 26, and 30 innervate dendritic spines (s), whereas Boutons 9 and 24 innervate dendritic shafts (d). All targets, except the dendrite in F are innervated by additional boutons (b) forming type-1 synapses (arrowheads). All scale bars represent 500 nm. MIT: mitochondrion; sn: spine neck. k–l) Electron micrograph (k) and 3D reconstruction (l) of the axon with 2 boutons (B25, B26, yellow) and their target spiny pyramidal cell dendrite (PYR, green). The target of B25 (s1) is a stubby spine (see G), that of B26 (s3) is a mushroom spine (see H). Two other spines were also identified (2, 4). All 4 spines receive a type 1 synapses (magenta). m) Proportion of synaptic targets (n, shown on top) of 4 CB+ DBCs (cell numbers as in Fig. 1).

In order to explore the synaptic targets of the “horsetail” axons, we used serial section electron microscopy of individual DBCs’ axonal boutons and compared their distributions to the synapses made by PV-DTCs, which have a very different axonal shape. We studied the “horsetail” axons of 4 CB+ DBCs (5, 10, 22, and 23), and 1 CR+ DBC (25). All samples were taken from layer III and included small collaterals (Fig. 6a). All CB+ DBCs examined were recorded in the temporal association cortex, whereas the CR+ DBC was recorded in the inferior parietal lobule. One DBC (5) was recorded in a sample from an epilepsy patient, and the rest from tumor patients (see Table 2).

Synaptic junctions were identified based on well-established electron microscopic criteria. These included the electron opaque synaptic cleft, a regular and uniform apposition of the presynaptic and postsynaptic membranes, the slight widening of the extracellular space, accumulation of synaptic vesicles at the presynaptic active zone, and the presence of a varying width of postsynaptic density (Fig. 6e–j). We examined a total of 132 boutons (20–34 from each DBC), of which 100 were found to form synaptic junctions. For 32 boutons, the synaptic junctions could not be identified because of an incomplete series of sections or because of the unfavorable cutting angle relative to the synaptic membranes, and 3 boutons formed 2 separate synaptic junctions each, all other boutons (97%) formed just 1 synapse, thus the total number of identified synaptic junctions was 103. In 9 instances, the synaptic junction was identified, but the small diameter target structure could not be defined, so a total of 94 synaptic targets were identified. All synapses made by DBCs were of Gray’s type II or symmetric (Gray 1959; Colonnier 1968) with a thin if any postsynaptic density (Fig. 6e–j), consistent with the GABAergic nature of DBCs shown here. The shape and size of vesicles could not be studied in the labeled boutons, being covered by the electron opaque DAB/OsO4 precipitate (Fig. 6e–j). All examined synaptic junctions were made with dendritic spines or dendritic shafts; no synapse was found on soma. Small diameter processes of triangular or elongated shapes and without mitochondria were identified as spines if the process could be followed to its end in 1 direction through serial sections (e.g. Fig. 6e, g, h, j; total no. of spines: 39). Some spines (33%) contained a spine apparatus, a membranous structure with multiple cisternae (Fig. 6h). All dendritic spine targets were also innervated by an unlabeled type I (asymmetric) synapse (Fig. 6e, g, h, j). We followed spine targets through serial sections in an attempt to identify the parent dendritic shaft to predict its origin from an interneuron or a pyramidal cell. Due to loss of sections and the very thin and long shape of some of the spine necks, only 9 spines could be traced back to their parent shafts. Three of these dendritic shafts emitted spines other than that innervated by the DBC and none of the dendrites received type I synapses on their shaft, thus most likely belonged to pyramidal cells. Nevertheless, given the abundance of dendritic spines on pyramidal cells relative to interneurons, it is likely that the majority of spine targets belong to pyramidal cells. In the case of the CB+ DBC 5, one postsynaptic dendrite was innervated by 2 labeled boutons, both of them targeting spines. These spines also received a type I synapse each. Two other spines on the same target dendrite received a single type I synapse each (Fig. 6k and l).

Dendritic shafts postsynaptic to DBCs could not be followed to an end in either direction and contained mitochondria and/or microtubules among other organelles (Fig. 6f and i). We identified a total of 55 dendritic shaft synaptic targets and followed them through serial sections. Of these, 10 had at least 4 (average: 6.4 ± 2.6) type I and type II additional synapses covering their surface densely. As no spines originated from these dendrites, we identified them as belonging to GABAergic interneurons. We grouped 23 dendritic shaft targets with at least 3 innervating synapses (including the labeled synapse) and lacking spines as originating from GABAergic interneurons. On 32 dendritic shaft targets, either no or only one additional synapse could be found due to short and incomplete series of sections; 3 of these also emitted spines. These 3 dendrites were grouped as pyramidal cell dendrites, whereas 29 postsynaptic dendritic shafts remained unidentified. The average diameter of the target shafts was of median 623, IQR: 454-nm diameter, and some were of very small diameter (e.g. Fig. 6f), corresponding to distal dendrites.

Among the CB+ DBCs, the average proportion of dendritic spine targets was 48 ± 15% (Fig. 6m; DBCs 5, 10, 22, and 23, *n* = 23, 22, 18, and 13, respectively), the remainder (52 ± 14%) being dendritic shafts. The CB−/CR+ DBC 25 had the lowest proportion (17%) of dendritic spine targets (3/18), the majority of postsynaptic elements (83%) being dendritic shafts. The frequency distribution of synaptic targets was different between all DBCs, including the CB−/CR+ DBC 25 (*X*^2^  _(4, 94)_ = 11.31, *P* = 0.023), meaning that the proportion of shaft and spine targets depends on the identity of the DBCs. When looking at only the CB + DBCs, no such dependence was observed (*X*^2^  _(3, 76)_ = 5.51, *P* = 0.138). Among the targets of CB+ DBCs identified as originating from GABAergic interneurons or pyramidal cells, the average proportion of pyramidal cell targets was 76.5 ± 18%, whereas that of identified interneuron targets was 23.5 ± 18% (DBCs 5, 10, 22, and 23, *n* = 17, 11, 11, and 6, respectively). On the other hand, the proportion of pyramidal cell targets for the CB−/CR+ DBC No 25 was only 21.4%, the majority of the targets (78.6%) being interneurons. These synaptic target distributions are similar to what was reported in other species (Somogyi and Cowey 1981; DeFelipe et al. 1989; DeFelipe and Jones 1992; Tamás et al. 1997a), but the homology of cell types is difficult to establish (see Discussion).

**Fig. 7:**
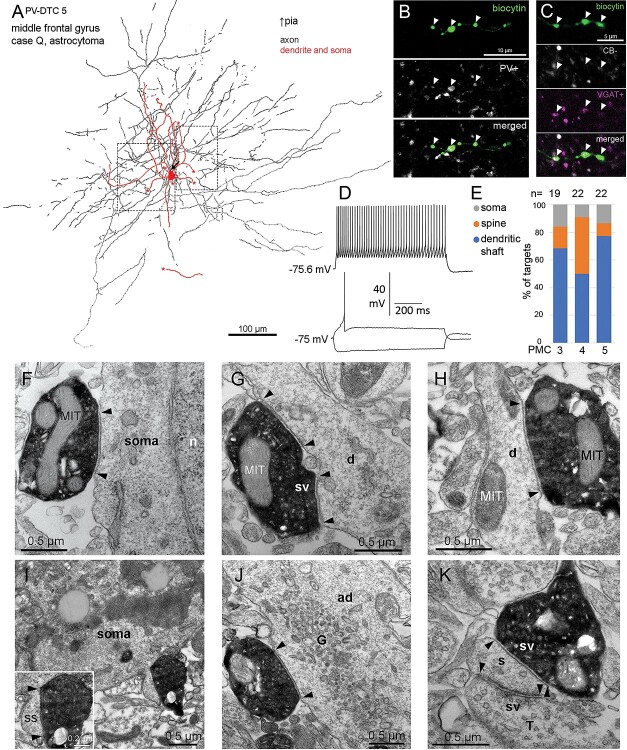
Synaptic targets of PV-DTCs in layer III of the human neocortex. a) Partial 2D reconstruction from 2 sections of ~60-μm thickness of PV-DTC 5 with dendrites (red) and axons (black) extending in every direction uniformly around the cell body in layer III. The AIS (arrow), cut ends of dendrites (asterisks), and the direction of the pial surface are indicated. Boxes indicate the areas re-embedded for electron-microscopy. b) Boutons (arrowheads) of the cell in a are immunopositive for PV (white) (single optical slice of 0.4-μm thickness). c) Boutons (arrowheads) of the cell are immunopositive for vesicular GABA transporter (VGAT, magenta) and immunonegative for CB (white) (single optical slice of 0.4-μm thickness). d) Voltage responses of the cell held in current-clamp mode to current injections of rheobase, holding current −100 pA, and rheobase +100 pA. e) Proportion of synaptic targets for PV-DTCs 3, 4, and 5 (*n* = 19, 22, and 22, respectively), as identified by serial section electron microscopy. f–k) Electron micrographs of labeled boutons (black, electron opaque) of PV-DTC 5 (f–h) and PV-DTC 4 (i–k), forming type II synaptic junctions with somata (e, i), inset in i shows the bouton innervating a somatic spine (ss), dendritic shafts (d, ad; g, h, j), and a dendritic spine (s, k). Edges of the synaptic junctions are indicated by arrowheads. The spine in k also receives a type I synapse (arrowheads) from an unlabeled terminal (T). n: nucleus, MIT: mitochondrion, d: dendritic shaft, ad: apical dendrite; ss: somatic spine, sv: synaptic vesicles, G: Golgi apparatus.

We also studied a total 104 boutons from 3 PV-DTCs (3, 4, and 5) and identified 68 synaptic junctions with 63 identified postsynaptic structures (Fig. 7). For 36 boutons, the synapse could not be found due to incomplete series of sections or unfavorable cutting angle; in the case of 5 synapses, the postsynaptic structure could not be defined. PV-DTCs 3 and 4 were recorded in the temporal cortex, whereas PV-DTC 5 in the frontal cortex. All samples came from tumor patients. All synapses made by PV-DTCs were of Gray’s type II (Fig. 7f–k), but in contrast to DBCs, PV-DTCs also targeted somata in addition to dendrites. Based on the presence of the nucleus (Fig. 7e) or on the size of the profile, a total of 8 targets were identified as soma; 4 of the somata were identified as pyramidal cells, based on the presence of only additional type I synapses. We identified 41 targets as dendritic shafts (Fig. 7g, h and j), of which 5 were identified as interneuron dendrites (Fig. 7h), and 36 as pyramidal cell dendrites (Fig. 7g and j), based on the same criteria as above. Finally, 13 targets were identified as dendritic spines (Fig. 7k), of which 6 contained a spine apparatus. One soma was found to be innervated by 2 boutons of a single PV-DTC. Interestingly, PV-DTC 4 had a higher proportion of spine targets (40%) compared with the other 2 (13.3 and 12.5% for PV-DTC 3 and 5, respectively; Fig. 7e). The average frequency of soma targets was 12.8 ± 3.4% for PV-DTCs, whereas that of dendritic shafts and spines was 65.2 ± 13.9% and 21.9 ± 16.7%, respectively. When comparing this with the target frequency distributions of CB+ DBCs, we found that there was significant correlation between the cell type and the frequency of different synaptic targets (*X*^2^  _(12, 126)_ = 28.066, *P* = 0.005); thus, CB+ DBCs and PV-DTCs have different synaptic target preferences in the human neocortex. Although PV-DTCs innervate somata to some extent, the majority of their synapses are onto dendrites, similarly to DBCs. To test whether different dendritic domains are innervated by these 2 cell types, we measured the diameters of target dendrites. We found no difference in the diameter of target dendrites innervated by DBCs and PV-DTCs (DBCs median: 623, IQR: 454 nm vs. PV-DTCs median: 726, IQR: 550 nm, Mann–Whitney U-test U = 378, *Z* = −1.43, *P* = 0.153), suggesting that DBCs and PV-DTCs innervate overlapping dendritic domains.

We conclude that CB+ DBCs have different synaptic target preference from the CB− DBC and PV-DTCs in the human neocortex, and they dedicate a high proportion of their synapses to dendritic spines of pyramidal cells. Although PV-DTCs also innervate dendritic spines, the majority of their targets are dendritic shafts and they also innervate somata, which DBCs avoid. The dendritic domains targeted by DBCs and PV-DTCs overlap.

### Targets of GABAergic synapses in the neuropil of layer III

DBCs and PV-DTCs significantly differ in their output postsynaptic target distributions. In order to compare their selection of output synapse locations to the distribution of potential GABAergic synaptic target sites in the neuropil, we have studied the distribution of GABAergic synapses on dendritic spines and shafts in layer III. Using postembedding immunogold reactions with antibodies to GABA in electron-microscopic sections (Somogyi and Soltész 1986) and a stereological method based on 3D tracing of synapses and their targets in a defined volume through serial sections (West 1999; Cano-Astorga et al. 2021), we determined the numerical density and the proportion of GABAergic synapses and their neuropil targets in samples from temporal and frontal cortices.

**Fig. 8:**
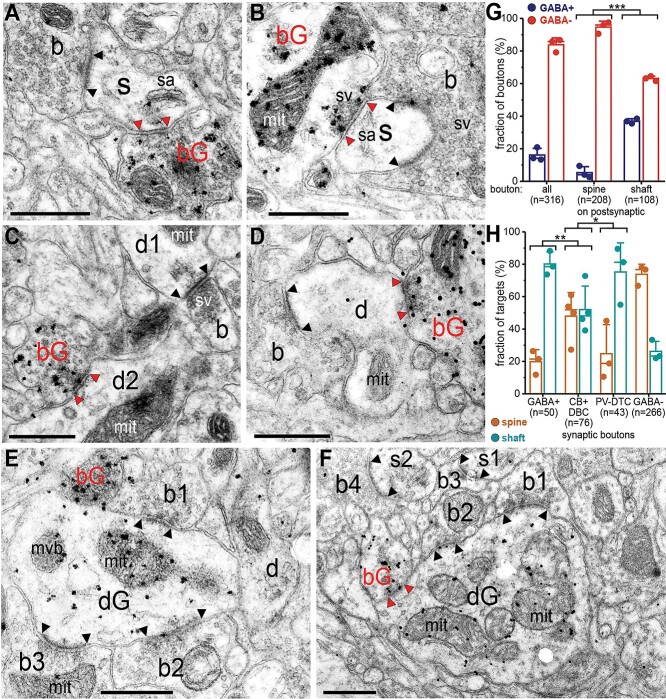
Synaptic targets of GABAergic and non-GABAergic synapses in the neuropil. a–f) Electron micrographs of sections immunolabeled for GABA with silver-enhanced colloidal gold particles (electron opaque dots). Gold particles are enriched over the surface of GABA-positive boutons (bG) and some dendrites (dG). a and b) Double innervation of dendritic spines (s) with spine apparatus (sa) by a GABA+ bouton (bG) and another bouton not labeled for GABA (b). Some synaptic vesicles (sv) are indicated in the presynaptic boutons. c) A GABA+ bouton (bG) innervating a GABA-negative dendritic shaft (d2), identified by the presence of a mitochondrion (mit). A GABA-negative bouton (b) innervates a different GABA-negative dendritic shaft (d1). d) A GABA+ (bG) and a GABA-negative bouton (b) innervate the same GABA-negative dendritic shaft (d). e) Three GABA-negative boutons (b1–3) form type I synaptic junctions with a GABA+ dendritic shaft containing a multivesicular body (mvb); another GABA+ bouton (bG) is also in contact with the dendrite but a synaptic junction is not visible. A different dendrite not labeled for GABA (d) is present. f) Two GABA-negative (b1–2) and a GABA+ boutons (bG) innervate a GABA+ dendritic shaft (dG). Two other GABA-negative boutons (b3–4) innervate a dendritic spine each (s1–2). Note the large number of mitochondria present in the target GABA+ dendrite. The synaptic specializations are indicated between arrowheads (red for GABAergic, black for non-GABAergic synapses). Scale bars in a–f, 0.5 μm. g) Fraction of GABA+ and GABA-negative (GABA-) synaptic boutons innervating dendritic spines or shafts (*X*^2^_(1, 316)_ = 55.44, *P* = 9.6*10^−14^ between spine and shaft targeting synapses). h) Fraction of dendritic spine and shaft synaptic targets innervated by different populations of presynaptic boutons, including those from physiologically recorded and labeled DBCs and PV-DTCs (*X*^2^_(1, 126)_ = 9.75, *P* = 0.0018 between GABA+ and DBC boutons; *X*^2^_(1, 119)_ = 5.46, *P* = 0.0195 between DBC and PV-DTC boutons). Bars: mean, whiskers: one standard deviation, line: median, number of boutons are indicated, individual data points are shown.

Boutons were identified as GABAergic based on the enrichment of silver-intensified gold particles over their cross-sectional surface consistently from section to section (Fig. 8a–f). Enrichment of gold particles was present over mitochondria in GABAergic postsynaptic dendrites (Fig. 8e and f) as well, but this signal was usually weaker than in boutons. Boutons making type I synapses, dendritic spines, spiny dendrites, and most dendritic shafts only had a low level if any particles which we considered technical background (Fig. 8a–d). Boutons immunopositive for GABA (GABA+) established typical Gray’s type II synapses with thin postsynaptic densities and targeted mostly dendritic shafts (Fig. 8c–f) and less frequently dendritic spines (Fig. 8a and b). All but 2 of the GABA+ bouton innervated dendritic spines received an additional type I synapse from a GABA-negative bouton. Most boutons not labeled for GABA (GABA−) established typical type I synapses with thick postsynaptic densities (Fig. 8a–f) and targeted mostly dendritic spines.

We counted a total of 316 synapses in a volume of 252.5 μm^3^ of temporal cortex (*n* = 4 3D probes, case AA) and 176.4 and 238.5 μm^3^ of frontal cortices (*n* = 5 3D probes, case BB; *n* = 2 3D probes, case CC; volumes are corrected for tissue compression along the cutting direction, but not for tissue shrinkage during dehydration). The mean numerical density of synapses across all 3 samples was 0.48 ± 0.04 per μm^3^ of neuropil, which excluded cell bodies, and appeared lower in the temporal (0.42) than in the frontal (0.52 and 0.50) cortex. The fraction of synapses made by GABA+ boutons was 16.1 ± 3.4% (Fig. 8g; mean density: 0.08 ± 0.02 per μm^3^; *n* = 3 patients). Dendritic spines were the targets of the majority of synapses (65.4 ± 5.2%), and only 5.3 ± 3% of these were made by GABA+ boutons (Fig. 8g). The proportion of spines receiving double innervation by one GABA+ and one GABA− bouton to those receiving at least 1 GABA− synapse was 4.6 ± 2.7%. In contrast, the proportion of GABA+ boutons among those targeting dendritic shafts was significantly higher (36.9 ± 1.2%; Fig. 8g; *X*^2^_(1, 316)_ = 55.44, *P* = 9.6*10^−14^ for the proportion of GABA+ boutons among those targeting dendritic spines vs. those targeting dendritic shafts). Among the dendritic shafts that received synapses, 9.3 ± 5.4% were GABA-immunopositive. Finally, the majority of GABA+ boutons (80.2 ± 6%) targeted dendritic shafts (Fig. 8h), whereas the majority of boutons not labeled for GABA (73.8 ± 5.1%) targeted dendritic spines (Fig. 8h).

There was a significant association between the proportion of dendritic spine to dendritic shaft targets and the different populations of synaptic bouton (*X*^2^_(3, 435)_ = 81.49, *P* = 0, Fig. 8h). Specifically, a significantly higher proportion of dendritic spine targets was found among DBC boutons than among all GABA+ boutons (*X*^2^_(1, 126)_ = 9.75, *P* = 0.0018) or among PV-DTC boutons that targeted spines or shafts (excluding those innervating somata) (*X*^2^_(1, 119)_ = 5.46, *P* = 0.0195). The proportion of dendritic spine targets was not different between PV-DTC boutons and all GABAergic boutons (*X*^2^_(1, 93)_ = 0.41, *P* = 0.521). Taken together, we have observed that DBCs but not PV-DTCs selectively target dendritic spines as postsynaptic elements.

### Regulation of GABAergic inputs to DBCs and PV-DTCs by group III mGluRs

We have shown that in the human neocortex, 2 distinct types of GABAergic interneuron, DBCs and PV-DTCs, have distinct axonal and dendritic distributions and innervate different subcellular compartments of their target cells. Furthermore, differences in the dendritic distribution and passive and active membrane properties between DBCs and PV-DTCs suggest that they receive and process synaptic inputs differently. The synaptic inputs of DBCs are not known. Group III metabotropic glutamate receptors (mGluRs) are presynaptic regulators of both GABAergic and glutamatergic synaptic transmission in many distinct forebrain neuronal circuits in rodents. Synaptic pathway- and cell type-specific expression of group III mGluRs in the rodent cortex (Shigemoto et al. 1996; Dalezios et al. 2002; Somogyi et al. 2003; Ferraguti et al. 2005) is thought to result in the differential inhibition of GABAergic synaptic transmission to different types of interneuron and pyramidal cells (Semyanov and Kullmann 2000; Kogo et al. 2004). However, neither the subcellular localisation nor the functions of group III mGluRs are known in the human neocortex.

We tested if GABAergic synaptic transmission to different types of interneuron in the human neocortex is differentially regulated by group III mGluRs. Specifically, we tested if activation of group III mGluRs by the orthosteric agonist *L-2-amino-4-phosphonobutyric acid* (L-AP4) depresses the frequency and/or amplitude of GABA_A_ receptor mediated sIPSCs, as has been shown in rodents (Mitchell and Silver 2000; Giustizieri et al. 2005; Cuomo et al. 2009). It is important to note that APs and fast glutamatergic synaptic transmission were not blocked in these experiments.

**Fig. 9:**
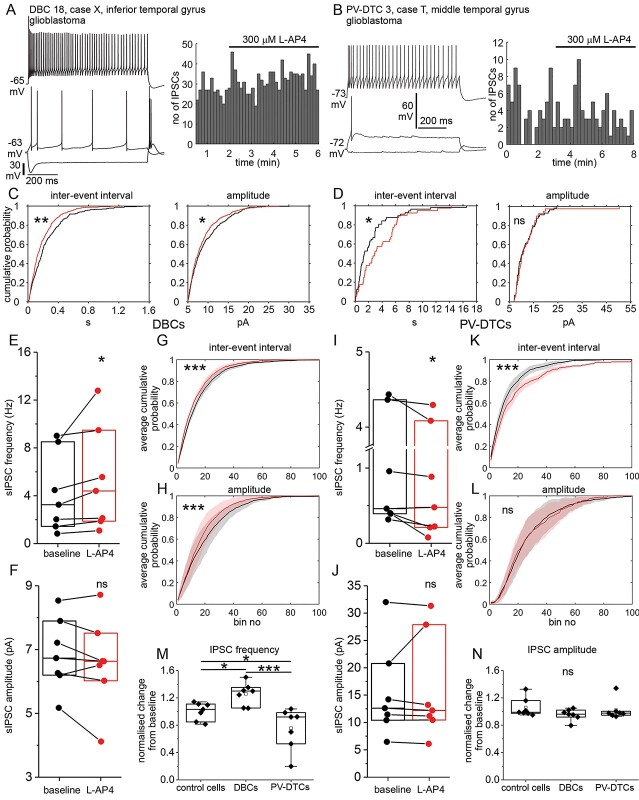
Effects of group III mGluR activation on sIPSCs in DBCs and PV-DTCs. a) Effect of group III mGluR activation on a DBC. Left: Voltage responses of DBC 18 to current injections of RB, RB +100 pA, and holding current −100 pA; right: event time histogram of sIPSCs recorded from DBC 18 during baseline and bath application of 300 μM L-AP4 (*n* = 1326 events, bin width: 8 s). b) Effect of group III mGluR activation on a PV-DTC. Left: Voltage responses of PV-DTC 3 to current injections of RB, RB +200 pA, and holding current −100 pA; right: event time histogram of sIPSCs recorded from PV-DTC 3 during baseline and bath application of 300 μM L-AP4 (*n* = 140 events, bin width: 10 s). c) Cumulative probability distribution of sIPSC interevent intervals (left) and amplitudes (right) in baseline (black, *n* = 326) vs. L-AP4 (red, *n* = 528) conditions in DBC 18 (Kolmogorov–Smirnov test, *P* = 0.001 for intervals and *P* = 0.0466 for amplitudes). d) Same as c for PV-DTC 3 (baseline, black, *n* = 59 vs. L-AP4, red, *n* = 41; Kolmogorov–Smirnov test, *P* = 0.048 for intervals and *P* = 0.876 for amplitudes). e) Mean sIPSC frequency of DBCs in baseline vs. L-AP4 conditions (paired sample Wilcoxon signed rank test *W* = 0, *Z* = −2.282, *P* = 0.016, boxes represent median and IQR). f) Median sIPSC amplitude of all DBCs in baseline vs. L-AP4 conditions (paired sample Wilcoxon signed rank test *W* = 22, *Z* = 1.268, *P* = 0.219). g) Mean cumulative probability distribution (line) and 95% CI (shaded areas) of sIPSC interevent intervals across all DBCs in baseline (black) vs. L-AP4 (red) conditions (100 bins/cell, Kolmogorov–Smironov test, *P* = 4.8 * 10^−5^). h) Average cumulative probability distribution of sIPSC amplitudes across all DBCs (100 bins/cell, Kolmogorov–Smironov test, *P* = 3.2 * 10^−4^). i–l)Same as e–h for PV-DTCs (i, j: paired sample Wilcoxon signed rank test *W* = 27, *Z* = 2.113, *P* = 0.031 for intervals; *W* = 20, *Z* = 0.93, *P* = 0.375 for amplitudes; k, l: Kolmogorov–Smirnov test, *P* = 5.7 * 10^−10^ for intervals and *P* = 0.099 for amplitudes). m and n) Normalized change from baseline to L-AP4 in sIPSC frequency (m) and amplitude (n) in control cells (*n* = 7), DBCs (*n* = 7), and PV-DTCs (*n* = 7) (Kruskal–Wallis ANOVA test, for frequency, *P* = 0.002, Conover post hoc test, adjusted by the Holm FWER method, *P* = 0.011 for controls vs. DBCs, *P* = 0.043 for controls vs. PV-DTCs, *P* = 0.0001 for DBCs vs. PV-DTCs; for amplitude, *P* = 0.382, boxes represent median and IQR, square symbol, mean, whiskers, and SD).

We recorded sIPSCs in whole-cell voltage clamp, at just below firing threshold potential (−50 to −45 mV), using a low Cl^−^ (4 mM) concentration in our internal pipette solution. In this setting, sIPSCs presented as upward deflections in the holding current with a fast rising phase and an exponential decay (Figs. 1c and 4c). In this experimental configuration, we also recorded AMPA receptor mediated spontaneous excitatory postsynaptic currents (sEPSCs, Fig. 1c), but these were not analyzed. We recorded sIPSCs in a total of 185 neurons and tested the effects of L-AP4 on 133, of which 65 were analyzed, including 49 putative interneurons and 16 putative pyramidal cells. The remainder 68 cells did not pass technical criteria to be included in the study (see Materials and methods). After microscopic examination of the visualized and pharmacologically tested cells, we identified 7 DBCs (see Fig. 1), 7 PV-DTCs (see Fig. 5), 2 neurogliaform cells, 1 rosehip cell (Boldog et al. 2018), and 1 axo-axonic cell, based on the specific distribution of their axon. The cell type identity of 29 interneurons could not be determined from their axonal and dendritic patterns, and 2 cells were not recovered, and thus, these were not included in this study. Four pyramidal cells were confirmed visually, based on their densely spiny dendrites. We compared the effects of L-AP4 on sIPSCs in DBCs and PV-DTCs as these 2 groups had enough identified cells to study the effects at the population level (Fig. 9). To control for spontaneous rundown of synaptic activity in vitro, and variability in slice conditions and technical parameters, the effects were compared with vehicle-treated control cells (*n* = 7), including 4 interneurons and 3 pyramidal cells. Median sIPSC frequency was 2.0 Hz (IQR: 3.6 Hz, *n* = 18) in interneurons and 1.6 Hz (IQR: 1.3 Hz, *n* = 16) in pyramidal cells, whereas median amplitude was 9.0 pA (IQR: 5.9 pA) in interneurons and 12.3 pA (IQR: 5.7 pA) in pyramidal cells.

In 3 out of 7 DBCs, bath application of L-AP4 (50 μM, *n* = 3; or 300 μM, *n* = 4) unexpectedly shifted the cumulative distribution of sIPSC interevent intervals to the left (e.g. Fig. 9c left, 2 sample Kolmogorov–Smirnov test, *P* = 0.001, *n* = 854 IPSCs), reflecting that the proportion of shorter interevent intervals increased compared with longer ones, corresponding to an increase in the frequency of sIPSCs. An increase in the number of sIPSCs after application of L-AP4 can also be seen on the event time histogram for DBC 18 (Fig. 9a). In the other 4 DBCs, the interevent interval did not change. However, the leftward shift in the distribution of sIPSC intervals was still present when averaged across all DBCs (Fig. 9g, 2 sample Kolmogorov–Smirnov test, *P* = 4.8 * 10^−5^, *n* = 100 bins). At the population level, the frequency of sIPSCs increased from median 3.25 Hz (IQR: 7.08 Hz) at baseline to median 4.4 Hz (IQR: 7.6 Hz; Wilcoxon signed rank test *W* = 0, *Z* = −2.282, *P* = 0.016, *n* = 7; Fig. 9e). The increase in frequency was not accompanied by a change in amplitude (Fig. 9f, baseline median: 6.7, IQR: 1.7 pA vs. L-AP4 median: 6.6, IQR: 1.5 pA, Wilcoxon signed rank test *W* = 22, *Z* = 1.268, *P* = 0.219), although in individual DBCs L-AP4 shifted the cumulative distribution of sIPSC amplitudes to the left (smaller amplitudes, e.g. Fig. 9c right, 2 sample Kolmogorov–Smirnov test, *P* = 0.047, *n* = 854 IPSCs) in 4 cells; to the right (larger amplitudes) in 1, and did not change in the remaining 2. The average cumulative distribution of sIPSC amplitudes also shifted toward smaller amplitudes (Fig. 9h, 2 sample Kolmogorov–Smirnov test, *P* = 3.2 * 10^−4^, *n* = 100 bins). Washout of L-AP4 from the bath did not lead to the recovery of the baseline frequencies or amplitudes. These results are in contrast with previous studies in rodents, where L-AP4 suppressed spontaneous and evoked IPSCs (Gerber et al. 2000; Matsui and Kita 2003; Drew and Vaughan 2004; Lu 2007; Cuomo et al. 2009).

In contrast to DBCs, in PV-DTCs, bath application of 300-μM L-AP4 shifted the cumulative distribution of sIPSC interevent intervals to the right, corresponding to lower sIPSC frequency (e.g. Fig. 9d left, 2 sample Kolmogorv–Smirnov test, *P* = 0.048, *n* = 100 IPSCs) in 2 out of 7 cells, also seen as a decrease in the number of sIPSCs after application of L-AP4 in the event time histogram of PV-DTC 5 (Fig. 9b). In 5 cells, the distribution did not change. However, averaged across all PV-DTCs the distribution of sIPSC intervals shifted to the right, upon application of L-AP4 (Fig. 9k, 2 sample Kolmogorov–Smirnov test, *P* = 5.7 * 10^−10^, *n* = 100 bins). At the population level, although the median frequency increased from 0.46 Hz (IQR: 4 Hz) in baseline to 0.48 Hz (IQR: 3.9 Hz), the pairwise decrease was significant (Fig. 9i, Wilcoxon signed rank test *W* = 27, *Z* = 2.113, *P* = 0.031). Neither did the cumulative distribution of sIPSC amplitudes change upon application of L-AP4 (Fig. 9l, 2 sample Kolmogorov–Smirnov test, *P* = 0.099, *n* = 100 bins), nor did the median amplitude at the population level (Fig. 9j, baseline median: 12.6, IQR: 10.4 pA vs. L-AP4 median: 12.2, IQR: 17.4 pA, Wilcoxon signed rank test *W* = 20, *Z* = 0.93, *P* = 0.375, *n* = 7). These effects are in agreement with results in rodents (Gerber et al. 2000; Matsui and Kita 2003; Drew and Vaughan 2004; Lu 2007; Cuomo et al. 2009).

We further assessed whether the changes measured upon L-AP4 application are different from spontaneous changes in frequency and amplitude due to changing slice conditions, technical parameters, and synaptic rundown. We compared the baseline normalized changes measured in DBCs and PV-DTCs to the changes measured in vehicle-treated control cells (Fig. 9m and n). The median change in sIPSC frequency was to 103% (IQR: 26%) of baseline in vehicle-treated controls (*n* = 7). This was significantly smaller than the change in DBCs (median: 130%, IQR: 30%, Conover post hoc test, adjusted by the Holm FWER method, *P* = 0.011 for controls vs. DBCs) and significantly larger than the change observed in PV-DTCs (median: 92%, IQR: 46%, Conover post hoc test, adjusted by the Holm FWER method, *P* = 0.043) (Fig. 9m). The changes were also different between DBCs and PV-DTCs (Conover post hoc test, adjusted by the Holm FWER method, *P* = 0.0001). Conversely, normalized change in sIPSC amplitude in DBCs and PV-DTCs did not differ from the change in vehicle-treated controls (Fig. 9n, Kruskal–Wallis test, *P* = 0.382). These comparisons show that L-AP4 alters the frequency rather than on the amplitude of sIPSCs, which indicates that L-AP4 primarily acts through modulation of neurotransmitter release at the level of axon terminals, in accordance with previous studies in rodents (Gerber et al. 2000; Semyanov and Kullmann 2000; Matsui and Kita 2003; Drew and Vaughan 2004; Kogo et al. 2004; Cuomo et al. 2009). However, in our experiments, APs and fast glutamatergic synaptic transmission were not blocked, thus such conclusions could not be drawn, and the unexpected increase of sIPSC frequency in DBCs is likely the result of a complex network effect, by group III mGluRs acting at both GABAergic and glutamatergic synapses and multiple levels of synaptic connections, resulting in the disinhibition of a GABAergic synaptic input specific to DBCs. Nevertheless, these results show a cell type-specific modulation of GABAergic synaptic input to 2 distinct types of GABAergic neuron in the human neocortex.

### Analysis of the effects of group III mGluR activation on sIPSC frequency with respect to patient-related variables

We showed differential effects of group III mGluR activation on sIPSCs in 2 different GABAergic neuron types on a small number of neurons which were recorded in samples of different cortical regions from patients with various diagnoses and medical history. Samples from tumor patients were taken as far from the MRI-imaged tumor mass as possible, and in deep tumors cortical tissue was not affected by infiltration, as assessed by MRI. However, some samples showed some degree of infiltration and/or oedema (Table 2). Furthermore, chemical signals may not be limited to structures directly infiltrated by the tumor mass. Particularly, seizures associated with brain tumors and epilepsy can spread through the entire neocortex. Because group III mGluRs have been associated previously with epilepsy and seizures in rodents, we tested if the effects of group III mGluR activation on sIPSC frequency measured here correlated with the clinical symptoms of patients, such as the presence or absence of seizures, antiepileptic medication, or other pathological processes related to sample quality, such as the presence of infiltration or oedema. We also tested if the measured effects depended on the cortical region where the sample was taken from and the age and sex of the patients (see Table 2).

We generated several multiple linear regression models, which included different sets of independent variables, to find the ones which best explain the variability in the data. However, due to the low number of pharmacologically tested neurons (*n* = 14 cells, 7 DBCs, 7 PV-DTCs) and the uneven frequency of the different cortical areas and pathologies, the predictive value of these models is limited. Almost all pharmacological response data came from patients between 50 and 66 years of age (mean: 57.5 ± 2.4 years) except for 1 outlier (age: 31 years). Furthermore, 9 (64%) cells were recorded in samples from glioblastoma patients, and none in samples from TLE patients. Similarly, 11 cells (79%) were recorded in the temporal, 2 (14%) in the frontal, and only 1 (7%) in the parietal cortex. Of the 7 models generated (Table 4), models 1 (Adj. *R*^2^: 0.47, *F* = 4.79, *P* = 0.026), 4 (Adj. *R*^2^: 0.75, *F* = 6.44, *P* = 0.019), 5 (Adj. *R*^2^: 0.83, *F* = 8.51, *P* = 0.027), 6 (Adj. *R*^2^: 0.88, *F* = 14.74, *P* = 0.002), and 7 (Adj. *R*^2^: 0.47, *F* = 3.93, *P* = 0.04) were significantly different from a constant. All models included the cell identity as a factor. Model 1 included the age and sex in addition to the cell identity, model 4 included the age, sex, cortical region, pathology, the presence of oedema in the sample, and seizures, and model 5 included all parameters, except hypertension as factors. Model 6 included age, sex, the presence of oedema, seizures, and treatments with anticonvulsive and steroid medication, but not the cortical region and the pathology, whereas model 7 only included age, sex, and hypertension in addition to the cell identity (Table 4). In models 1 and 7, the cell identity was the only factor significantly different from zero, contributing to the pharmacological response; however, these models explained less than half of the variance in the data (Table 4). In model 5, seizures were a significant factor in addition to the cell identity. Interestingly, in model 4, all factors, except age and pathology, were significant. In model 6, in addition to the cell identity and age, seizures and anticonvulsive medication were significant factors with seizures adding to the increase in sIPSC frequency induced by L-AP4 (0.67 ± 0.14, *P* = 0.003), and anticonvulsive treatment having the opposite effect (−0.6 ± 0.17, *P* = 0.013; Table 4). Model 6 also explained the most in the variability of the change in sIPSC frequency (Adj. *R*^2^: 0.88). In all the different models, the most consistently significant factor was the cell identity; however, seizures were also significant in 3 models. Although these models support the conclusion that the effect of group III mGluR activation on sIPSC frequency is cell type-specific, considering the small number of data points, it cannot be excluded that other factors, particularly seizures and antiepileptic medication, might also have an effect on the measured responses.

**Table 4:** Multiple linear regression models for the effect of group III mGluR activation on sIPSC frequency.

Factor	Model 1	Model 2	Model 3	Model 4	Model 5	Model 6	Model 7
intercept	0.92 ± 0.46 *P* = 0.076	0.92 ± 0.49 *P* = 0.093	1.02 ± 0.47 *P* = 0.061	0.36 ± 0.5 *P* = 0.504	0.27 ± 4.72 *P* = 0.957	1.43 ± 0.36 ***P* = 0.008**	0.74 ± 0.49 *P* = 0.161
DBC	0.45 ± 0.16 ***P* = 0.018**	0.44 ± 0.17 ***P* = 0.033**	0.46 ± 0.17 *P* = 0.0.25	0.86 ± 0.17 **p = 0.003**	0.58 ± 0.2 **p = 0.043**	0.65 ± 0.1 ***P* = 0.0007**	0.43 ± 0.16 ***P* = 0.024**
age	−0.002 ± 0.009 *P* = 0.864	−0.0009 ± 0.01 *P* = 0.926	−0.004 ± 0.009 *P* = 0.652	0.004 ± 0.009 *P* = 0.666	0.02 ± 0.15 *P* = 0.88	−0.02 ± 0.007 ***P* = 0.044**	0.003 ± 0.01 *P* = 0.743
sex	−0.13 ± 0.18 *P* = 0.477	−0.14 ± 0.19 *P* = 0.487	−0.16 ± 0.18 *P* = 0.403	−0.42 ± 0.15 ***P* = 0.03**	0.1 ± 0.68 *P* = 0.896	−0.18 ± 0.11 *P* = 0.154	−0.21 ± 0.19 *P* = 0.296
Temporal cort.	-	−0.03 ± 0.19 *P* = 0.864	−0.1 ± 0.19 *P* = 0.614	−0.4 ± 0.16 ***P* = 0.045**	−1.03 ± 3.66 *P* = 0.792	-	-
glioblastoma	-	-	0.23 ± 0.16 *P* = 0.2	−0.2 ± 0.17 *P* = 0.308	0.36 ± 0.81 *P* = 0.685	-	-
oedema	-	-	-	0.74 ± 0.26 ***P* = 0.028**	1.18 ± 3.3 *P* = 0.74	0.31 ± 0.13 *P* = 0.054	-
seizures	-	-	-	0.49 ± 0.17 ***P* = 0.03**	0.69 ± 0.16 ***P* = 0.013**	0.67 ± 0.14 ***P* = 0.003**	-
levetiracetam	-	-	-	-	−0.7 ± 0.35 *P* = 0.114	−0.6 ± 0.17 ***P* = 0.013**	-
dexamethasone	-	-	-	-	−1.39 ± 5.43 *P* = 0.811	0.26 ± 0.15 *P* = 0.129	-
hypertension	-	-	-	-	-	-	−0.17 ± 0.16 *P* = 0.311
Df	10	9	8	6	4	6	9
Residual sum of squares	0.65	0.65	0.52	0.19	0.08	0.09	0.58
*R* ^2^	0.59	0.59	0.67	0.88	0.95	0.95	0.64
Adj. R^2^	0.47	0.41	0.47	0.75	0.83	0.88	0.47
ANOVA F	4.79	3.25	3.27	6.44	8.51	14.74	3.93
*P*	**0.026**	0.065	0.067	**0.019**	**0.027**	**0.002**	**0.04**

For each independent variable (factor), its coefficient is given as mean ± SE and the *P*-values are listed. ANOVA statistics for each model are given at the bottom.

## Discussion

Diverse types of local interneurons with specialized roles, as reflected in their distinct molecular expression, intrinsic biophysical properties, and synaptic relationships provide most of the GABAergic innervation of the cerebral cortex (Kawaguchi and Kubota 1997; Somogyi et al. 1998; Gupta et al. 2000; Defelipe et al. 2013; Hodge et al. 2019). We have characterized 2 distinct GABAergic interneuron types in human association neocortex, which differ in calcium-binding protein expression, intrinsic membrane properties, synaptic output, and the group III mGluR-mediated regulation of their GABAergic inputs. The DBCs are a cell type in the upper layers of the neocortex of primates with a striking columnar “horsetail” axon (Ramón y Cajal 1899; Jones 1975; Valverde 1978; Somogyi and Cowey 1981; DeFelipe et al. 1989; DeFelipe et al. 1990; Lund and Wu 1997) with as yet no clear homologue in rodents (Ballesteros Yáñez et al. 2005). In contrast, PV-DTCs with widely distributed axons resembling neurons described as “basket cells” have been illustrated in rodents (Kawaguchi and Kubota 1998; Kawaguchi and Kondo 2002; Packer and Yuste 2011), carnivores (Meyer 1983; Somogyi et al. 1983; Meyer and Ferres-Torres 1984; Kisvarday et al. 1987), and primates (Hendry et al. 1989; Lund and Lewis 1993; Gabbott and Bacon 1996a; Lund and Wu 1997), including humans (Marin-Padilla and Stibitz 1974; Kisvárday et al. 1990; Szegedi et al. 2017; Bakken et al. 2021). In terms of synaptic outputs, human CB-expressing DBCs innervate dendrites but not somata and preferentially target dendritic spines. In contrast, PV-DTCs target mainly dendritic shafts and to a lesser extent somata and dendritic spines. These 2 cell types also differ in the response of their GABAergic inputs to the activation of group III mGluRs, presynaptic receptors that inhibit GABA and glutamate release in rodents (Mitchell and Silver 2000; Semyanov and Kullmann 2000; Kogo et al. 2004).

### Molecular diversity of cortical GABAergic neurons

Among the many molecular differences of GABAergic interneurons, the selective expression of calcium binding proteins characterizes the families of cell types in rodents (Cauli et al. 1997; Kubota et al. 2011; Rudy et al. 2011; Tasic et al. 2018; Yao et al. 2021) and primates (Lund and Lewis 1993; Condé et al. 1994; Gabbott and Bacon 1996b; Zaitsev et al. 2005), including humans (DeFelipe 1997; González-Albo et al. 2001; Varga et al. 2015; Hodge et al. 2019). Each calcium binding protein is expressed by several distinct cell types and individual neurons may express more than 1 calcium binding protein; hence on their own, they are insufficient to delineate functionally defined cell types. In our recorded interneuron population, CB and CR were found in separate individual DBCs, which were all immunonegative for PV. Conversely, PV-DTCs were positive for PV and negative for CB and CR, except for 1 cell, which was double immunopositive for PV and CB. The differential expression of PV, CB, and CR in the interneurons we studied is in agreement with the mutually exclusive expression of CB and PV in the neocortex of monkeys (DeFelipe et al. 1989; Gabbott and Bacon 1996a), but different from rodent neocortex, where a large population of GABAergic neurons including basket cells co-expresses CB and PV (Kubota and Jones 1993; Cauli et al. 1997; Kawaguchi and Kubota 1997; Hartwich et al. 2009). In humans, CB and PV are co-expressed in axo-axonic cells (del Río and DeFelipe 1997a).

Transcriptomic analysis in humans indicates 4 main GABAergic neuronal types in the middle temporal gyrus (MTG; Hodge et al. 2019) and 4 in the primary motor cortex (M1; Bakken et al. 2021) expressing mRNA for CB (CALB1), based on median RNA counts. Sporadic expression of CALB1 is also present in other transcriptomic clusters in a smaller fraction of cells, while the median expression of the cluster is zero. In the MTG, among those groups with cell bodies in layers II and III where DBCs are located, the 2 largest groups either co-express somatostatin (*Inh L1–3 SST CALB1*) or CCK (*Inh L2–6 LAMP5 CA1*), although the latter group is more frequently found in layers V and VI. In M1, GABAergic neuron clusters with the highest expression of CALB1 and cell bodies in layers II and III are *Inh L1–2 SST CCNJL*, *Inh L1–2 SST PRRT4*, and *Inh L1–2 SST CLIC6*, all of which co-express high levels of SST (Bakken et al. 2021). However, we only found 1 CB+ DBC to be immunoreactive for somatostatin, and none immunoreactive for CCK, which of course does not exclude some level of mRNA expression. Although in the cat visual cortex, CCK was suggested to be expressed in DBC-like GABAergic neurons (Freund et al. 1986), it is unclear if those were homologous to the DBCs in humans. Furthermore, patch-seq data indicate that transcriptomic cell types in mice corresponding to these human SST clusters mainly include upper layer Martinotti cells, “L2/3 PV-like cells” and “lower FS basket cells” (Hodge et al. 2019; Callaway et al. 2021). In M1, the cluster *Inh L5–6 LAMP5 CRABP1* co-expresses CB and CCK; however, the homologous mouse cluster *LAMP5 LHX6* corresponds to neurogliaform cells (Callaway et al. 2021). Finally, in the MTG, the CALB1 expressing cluster *Inh L1–6 PVALB SCUBE3* homologous to the mouse clusters of axo-axonic cells (Hodge et al. 2019) expresses CB together with PV. The corresponding cluster in M1 (*Inh L1–6 PVALB COL15A1*) does not express CALB1 (Callaway et al. 2021). In accordance with the co-expression of CB with PV in 1 PV-DTC described here, scattered expression of CALB1 is also found in the human MTG cluster *Inh L2–4 PVALB WFDC2*, which is homologous to clusters including fast-spiking basket cells in mice (Hodge et al. 2019; Callaway et al. 2021). In summary, we are unable to allocate a clear transcriptomic cell type to DBCs at present. Considering the high abundance of CB+ DBCs in humans, it is possible that they are vulnerable to cell sorting and are not represented in their real proportion in transcriptomic datasets. Alternatively, they may be included in the numerous *Inh L1–3 SST CALB1* group mixed with Martinotti cells, which have a very different axon. Our inability to detect somatostatin immunoreactivity in DBCs could be due to a genuine lack or very low levels of peptide expression. The same may apply to *Inh L2–6 LAMP5 CA1* having CCK peptide levels undetectable with our methods. Future patch-seq data in human cortex would clarify this issue.

Regarding PV-DTCs, in human MTG and M1, there are 2 and 4 PV-expressing transcriptomic cell types, respectively, with cell bodies in layers II and III (Hodge et al. 2019; Bakken et al. 2021). Of these, the *Inh L2–4 PVALB WFDC2* in MTG, and the *Inh L2–5 PVALB RPH3AL* or *Inh L3 PVALB SAMD13* in M1, are homologous to mouse transcriptomic cell types thought to include superficial-layer basket cells (Hodge et al. 2019; Gouwens et al. 2020; Callaway et al. 2021). However, because the synaptic target preference of the individual neurons mapped onto these transcriptomic clusters was not tested (Gouwens et al. 2020; Bakken et al. 2021), it is not known whether dendrite-targeting cells comparable to the PV-DTCs described here are represented in the same or different transcriptomic clusters.

The visualization of individual CB-positive DBCs proves that they contribute a major component of the CB immunoreactive radial axon bundles in humans and probably also in monkeys (del Río and Defelipe 1997; DeFelipe et al. 1989, 1990, 2006; Ferrer et al. 1992; Ballesteros Yáñez et al. 2005). Importantly, however, a radial axon bundle is likely to contain axons from multiple DBCs, including CB-immunonegative and CR-immunoreactive ones, and other CB-expressing interneuron types may also contribute. In many cortical areas, supragranular pyramidal cells express CB (Gabbott and Bacon 1996a; Kawaguchi and Kubota 1997), thus their axons may also be included. In carnivores, immunoreaction to CB also reveals axon bundles (Ballesteros Yáñez et al. 2005), but it is not clear if DBCs with horse tail axons contribute. Bundles of CB-positive axons have not been documented in rodents, lagomorphs, and ungulates (Ballesteros Yáñez et al. 2005). Calretinin-immunoreactive (CR+) bundles of axons were reported in humans (del Río and Defelipe 1997), but individual DBCs have not been previously visualized. Two of our recorded DBCs were immunopositive for CR and immunonegative for CB, but because of the small number of CR+ DBCs, we could not compare their features to the CB+ population. The available evidence from synaptic output of CR+ interneurons suggests that multiple cell types exist, some of which may specialize in innervating other interneurons (del Río and DeFelipe 1997c; Gabbott et al. 1997; Meskenaite 1997), in agreement with the synaptic target preference of the one CR+ DBC reported here and in contrast to the spine-preferring CB+ DBCs.

It is not yet clear whether DBCs are present in rodents, or if they are, whether they express CB. Neurons with descending axons, visualized and named DBCs in rats, do not have comparable “horsetail” axons to those in primates and express VIP, corticotropin releasing factor (CRF), and CR (Kawaguchi and Kubota 1996, 1997; Karube et al. 2004; Kubota et al. 2007). In transcriptomic studies, VIP is co-expressed with CB in only 1 group of cells (*L1–2 VIP WNT4*) in human M1 (Hodge et al. 2019; Bakken et al. 2021) (Allen Institute for Brain Science, 2021, Allen Cell Types Database—Human Multiple Cortical Areas dataset, available from celltypes.brain-map.org/rnaseq). Interneurons with descending “horsetail” axons and unknown molecular profiles were described in mice (Jiang et al. 2013, 2015), but another similar large scale study did not report such neurons (Gouwens et al. 2020). Furthermore, several other types of GABAergic interneuron in rodents, cats, and primates, particularly those expressing VIP and/or CCK, and/or CR (Freund et al. 1986; Condé et al. 1994; Kawaguchi and Kubota 1996, 1997; Meskenaite 1997; Caputi et al. 2009), and some basket cells (Somogyi et al. 1983; DeFelipe et al. 1986; Somogyi and Soltész 1986; Kisvárday 1992; Jiang et al. 2015) also have descending axon collaterals crossing several layers without forming a narrow bundle. In conclusion, the available evidence suggests that CB-positive DBCs are a primate specialization, and their preference for dendritic spine innervation (see below) is shared by homologous cell types in carnivores.

Parvalbumin-expressing interneurons include axo-axonic and basket cells in rats (Kawaguchi and Kubota 1998; Hartwich et al. 2009) and primates (Zaitsev et al. 2005), including humans (Szegedi et al. 2020). The term “basket cell” is often used loosely for PV-expressing multipolar cells without information if the cells so named form perisomatic baskets or terminate on somata at all. In all species, there is a high density of PV-positive synaptic boutons on cell bodies (Blümcke et al. 1991; Czeiger and White 1997; DeFelipe 1997), but they may be provided only by certain types of PV-expressing interneuron, the genuine basket cells. In the human cerebral cortex, such soma-preferring cells have not been visualized individually with their axons. The PV-DTCs recorded and visualized here targeted dendritic shafts and dendritic spines on proximal, as well as on distal dendrites with the majority of their synapses, whereas somata constituted only a small fraction of their targets. Similar dendrite-targeting GABAergic cells were also described in the frontal cortex of rats, some immunoreactive for PV (Kawaguchi and Kubota 1998), others for CB (Hartwich et al. 2009). Large, molecularly uncharacterized dendrite-targeting cells with similar synaptic target distribution were also reported in the visual cortex of cats (Somogyi and Soltész 1986; Tamás et al. 1997a) and shown to evoke different synaptic responses from those of synaptically defined basket cells (Tamás et al. 1997a).

The differential expression of CB and PV in DBCs and PV-DTCs, respectively, correlated with differences in their intrinsic firing properties, as described for neocortical GABAergic neurons in other species (Kawaguchi and Kubota 1993, 1996; Cauli et al. 1997, 2000; Zaitsev et al. 2005). The most common features of DBC voltage responses were the large amplitude voltage sag in response to hyperpolarizing current injection and spike amplitude accommodation in response to continuous depolarizing current injection. The amplitude of the voltage sag in DBCs is comparable to that of rosehip cells in humans (Boldog et al. 2018; Field et al. 2021); however, other yet undefined interneuron types, such as some expressing CB1 receptor along their axonal membrane, may also show similarly large voltage sag (Varga et al. 2015; Field et al. 2021). Surprisingly, neither neurons with radial axons expressing CB in the prefrontal cortex of monkeys (Zaitsev et al. 2005), nor neurons with radial axons recorded in the prefrontal cortex of rats named DBCs (Jiang et al. 2015) show these voltage patterns. This raises doubts about the homology between neurons called DBCs in different species. In addition to these features, some human DBCs also showed burst firing. In the rat prefrontal cortex, burst firing is most common in interneurons expressing neuropeptides such as VIP, CCK, and SM, including neurons in layers II and III, named DBCs based on their descending axonal arbors expressing VIP and\or CRF and\or CR (Kawaguchi and Kubota 1996; Karube et al. 2004). Martinotti cells also fire burst in layer V with ascending axonal arbors co-expressing SM and CB (Kawaguchi and Kubota 1996). Conversely, PV-DTCs had similar voltage sag and spike amplitude accommodation to PV-expressing fast-spiking neurons described previously in humans (Szegedi et al. 2017) and in other species (Kawaguchi 1995; Zaitsev et al. 2005; Povysheva et al. 2013). In our sample, there was no difference in the AP duration of DBCs and PV-DTCs, and both were shorter than that of regular-firing interneurons and longer than typical fast-spiking interneurons in other species (Kawaguchi 1995; Cauli et al. 1997; Krimer et al. 2005).

### Dendritic spines as GABAergic synaptic targets—contributions of DBCs and other interneurons

We have confirmed that dendritic spines are the targets of the majority of synapses in the neocortex (Rakic et al. 1986; Adams 1987; Beaulieu et al. 1992; Peters et al. 2008; Kasthuri et al. 2015; Domínguez-Álvaro et al. 2019; Cano-Astorga et al. 2021), and most of these are glutamatergic as predicted by the type I postsynaptic specialization. More than a quarter (26%) of such presumed glutamatergic synapses are on dendritic shafts of both pyramidal neurons and interneurons in humans. Only a small proportion of dendritic spines (~1.5–6%) are double innervated also by proven or presumed GABAergic synapses (Beaulieu et al. 1992; Kubota et al. 2007; Domínguez-Álvaro et al. 2018; Kwon et al. 2019; Cano-Astorga et al. 2021). Imaging studies of GABAergic synapses along a single pyramidal cell dendrite suggest a higher density and proportion of double innervated spines on distal than on proximal dendritic segments in mice (Chen et al. 2012). However, this might depend on the identity of the postsynaptic pyramidal cell.

The preferential innervation of dendritic spines by human DBCs confirms similar synaptic target selection in the cat (Tamás et al. 1997a; Tamas et al. 1998) and monkey (Somogyi and Cowey 1981). There is evidence that an individual DBC innervates both the dendritic spines (*n* = 6) and shafts (*n* = 4) of a given pyramidal cell in the same proportion as its overall postsynaptic target distribution in layer III (Tamás et al. 1997a), and both distal and proximal dendrites were innervated. The same is true for dendrite-targeting cells (Tamás et al. 1997a), which in addition to dendritic spines and shafts also innervate somata. We have also found evidence that multiple spines on the same pyramidal dendrite may be targeted by the same DBC, providing a focal input. The electron microscopic examination of CB+ axonal bundles in humans and monkeys (DeFelipe et al. 1989, 1990; DeFelipe and Jones 1992; del Río and DeFelipe 1995; Peters and Sethares 1997) also suggested that spines are extensively innervated, but the contribution of individual DBCs to the bundles or any bias in their target selection could not be established. There is some variability in the proportion of postsynaptic dendritic spine targets in the cat (38 and 69%), monkey (40%), and among the CB+ human DBCs (mean, 48%) studied here. Furthermore, 3 other Golgi impregnated interneurons with descending “horsetail” axons in cat visual cortex made much lower proportion of synapses on dendritic spines, the majority (~86%) of synaptic targets being dendritic shafts (Somogyi and Cowey 1981), similar to the CR+ cell reported here (83% dendritic shafts). In the absence of molecular data on these 3 cells, it is not clear if they were homologous to the DBCs in the monkey and humans. Although they were named DBCs based on their axonal patterns, these neurons may have been interneuron target preferring cell types, as a significant proportion of their synapses were found innervating nonpyramidal neuron somata and dendrites (Somogyi and Cowey 1981). Calretinin-expressing cells in other species frequently target interneurons (del Río and DeFelipe 1997c; Gabbott et al. 1997; Meskenaite 1997; Gonchar and Burkhalter 1999; Caputi et al. 2009).

The PV-DTCs studied here had similar synaptic target distribution to that of dendrite-targeting cells (Somogyi and Soltész 1986; Tamás et al. 1997a) in the visual cortex of the cat and to some fast-spiking PV-expressing interneurons in the frontal cortex of rats (Kawaguchi and Kubota 1998; Kubota et al. 2007). Others have also reported CB-expressing dendrite-targeting cells in the rat frontal cortex (Hartwich et al. 2009). Although the proportion of dendritic spine synaptic targets of PV-DTCs was significantly lower (~20%) than that of DBCs, and not different from the average proportion of GABAergic synapse targets in the human cortical neuropil, they may still make up a significant proportion of spine innervation depending on the abundance of this cell type, which is not known. Other GABAergic neuronal types, including Martinotti cells, neurogliaform cells, and basket cells, are also expected to contribute to dendritic spine innervation (Somogyi et al. 1983; Somogyi and Soltész 1986; Meskenaite 1997; Tamás et al. 1997a, 1997b, 2003; Kawaguchi and Kubota 1998; Kubota et al. 2007; Chiu et al. 2013). However, how dendritic spines receiving glutamatergic input from different sources are selected by GABAergic cell types remains unknown.

### Multiple, location-dependent effects of GABA in cortical circuits

The differential placement of GABAergic synapses along the postsynaptic pyramidal cell surface by DBCs and PV-DTCs in the human neocortex is most likely driven by a temporal division of labor between distinct types of GABAergic neuron, as most apparent in the hippocampus (Somogyi et al. 2014), but also evident in the neocortex (Somogyi et al. 1998; Hartwich et al. 2009; Massi et al. 2012; Averkin et al. 2016). For example, the GABAergic innervation of principal cells, which include, e.g. pyramidal cells, spiny stellate cells and dentate gyrus granule cells, along their axon initial segments (AISs) depolarizes spike initiation voltage threshold (Rojas et al. 2011), which is best achieved by concentrating all the synapses by axo-axonic cells to the AP initiation site. In contrast, the independent somatic GABAergic innervation decreases IR, shunting excitatory inputs (Rojas et al. 2011). This latter inhibitory function is best achieved by GABA distributed to multiple sites not only on the soma but also to the dendrites and even dendritic spines, as we have found for PV-DTCs and has been documented also for various basket cells (Kisvárday et al. 1985; Somogyi and Soltész 1986; Kawaguchi and Kubota 1998). Furthermore, perisomatic GABAergic innervation is well suited for controlling the timing of postsynaptic firing in rodents (Cobb et al. 1995; Miles et al. 1996; Tamás et al. 2000, 2004) and contributes to high-frequency network oscillations in vivo (Cardin et al. 2009). Conversely, experimental data and modeling indicate that dendrite-targeting GABAergic synapses modulate integration in distinct dendritic branches (Miles et al. 1996; Bloss et al. 2016), as has been shown for the rosehip cells in humans, which innervate the apical tufts of pyramidal cells in layer I (Boldog et al. 2018). On dendritic spines, GABAergic synapses should have an even more local effect on the glutamatergic terminal to the same spine. Indeed, these can selectively shunt penetration of somatically evoked Ca^2+^ signals into single dendritic spines and modulate local synaptic plasticity (Chiu et al. 2013). Soma- or dendrite-targeting interneuron types are differentially recruited by local pyramidal cells in both mouse and human neocortex (Obermayer et al. 2018). Nevertheless, GABAergic inputs to dendrites can also have a strong influence on postsynaptic firing through gating of dendritic spikes (Larkum et al. 1999; Lovett-Barron et al. 2012), and entraining firing rhythms at various frequencies (Szabadics et al. 2001; Tamás et al. 2004). The centripetally spreading shunting effect of multiple GABAergic synapses located on dendrites even distally to excitatory inputs may lead to the inhibition of AP generation (Gidon and Segev 2012).

The specific spatial distribution and target neuron selectivity, together with the intricate compartmentalized placement of GABAergic synapses on the surface of single neurons, can also lead to complex interactions with other inputs in the cortex (Varga et al. 2012; Somogyi et al. 2014). In this context, modeling suggests that GABAergic inputs on distal dendrites, where some of the DBC and PV-DTC synapses are located, may have an excitatory effect when arriving ahead in time to more proximally located glutamatergic input, even if E_GABA-A_ is negative to the AP threshold (Lombardi et al. 2021).

Spatial compartmentalization of molecular components involved in GABAergic signaling may further differentiate synaptic effects of individual presynaptic terminals. For example, a major determinant of intracellular Cl^−^ is the potassium chloride co-transporter, KCC2, that reduces intracellular Cl^−^. Interestingly, it is expressed at relatively high density in hippocampal pyramidal cell dendritic spines, particularly at the periphery of glutamatergic synapses (Gulyás et al. 2001), although its concentration along dendritic shaft membranes is higher (Báldi et al. 2010). Due to its diffusional isolation by the thin dendritic spine neck, even low expression of KCC2 would likely results in a negative E_GABA_ in dendritic spines and a hyperpolarising effect of fast GABA_A_ receptor signaling facilitated by the relatively small volume-to-surface ratio of spines. However, whether KCC2 expression is similar in human neocortical pyramidal neurons and in dendritic spines double innervated by GABAergic and glutamatergic synapses is not known. In any case, the GABAergic synapse made by the DBC is likely to have a major role on the plasticity of the glutamatergic synapse on the same spine and is well suited for an effective postsynaptic shunting or blocking of NMDA receptor-mediated currents and Ca^2+^-dependent synaptic plasticity (Chiu et al. 2013), as well as for a presynaptic inhibition of glutamate release through GABA-B receptors.

We have shown that DBCs and PV-DTCs are GABAergic but have not directly tested whether they were inhibitory. The postsynaptic effects of DBCs in unitary interactions seen here were consistent with fast phasic synaptic signaling mediated by GABA_A_ receptors, as shown previously in the cat (Tamás et al. 1997a). Phasic signaling involves fast and transient conductance through activated GABA_A_ receptors concentrated in the postsynaptic specialization in response to synaptic release of GABA (Mody et al. 1994; Nusser et al. 1997; Mozrzymas 2004; Farrant and Kaila 2007). The effect of synaptic GABA_A_ receptor activation on the postsynaptic membrane potential would depend on the reversal potential of GABA_A_ (E_GABA-A_) determined primarily by the local transmembrane gradient of Cl^−^ (Farrant and Kaila 2007), as well as on the timing of other inputs under physiological conditions. The DBC-evoked IPSCs recorded at the soma of postsynaptic neurons were outward currents, corresponding to a hyperpolarizing effect locally. The CB+ DBCs mainly innervated pyramidal cells, but 1 CR+ DBC mainly targeted interneuron dendrites. Selective inhibition of GABAergic neurons could lead to the disinhibition of their target neurons (Pi et al. 2013). Phasic signaling through GABA_A_ receptors may inhibit postsynaptic excitability and firing both by hyperpolarization and/or effectively shunting the inward currents generated by glutamate receptor activation particularly the slower NMDA receptor-mediated component (Dingledine et al. 1986; Collingridge and Bliss 1987; Staley and Mody 1992). Interacting with other time and voltage-dependent conductances, GABAergic phasic signaling may also change the spike timing of postsynaptic neurons without actually “inhibiting” the firing rate (Cobb et al. 1995).

Further selective effects could emerge from the differential allocation of synaptic and extrasynaptic GABA_A_ receptor species (Farrant and Nusser 2005; Field et al. 2021) as well as from the location of pre- and postsynaptic GABA_B_ receptors. Some GABAergic interneuron types, such as neurogliaform cells, have been shown to evoke both slow phasic and tonic GABA_A_ receptor signaling through extrasynaptic GABA_A_ and GABA_B_ receptors (Tamás et al. 2003; Oláh et al. 2007, 2009). The tight axonal bundles of DBCs form an exceptionally high-volume density of GABA-releasing boutons to which several individual cells contribute. If these neurons fired together, which may be facilitated by their mutual synaptic interactions as we show here, they would increase GABA concentration locally in a column that could lead to the activation of tonic currents and GABA-B receptors in dendrites and axons passing through such a column. Such a mechanism is unlikely to be involved in the action of PV-DTCs whose boutons are widely spread across several hundred microns of cortical tissue.

### Regulation of GABAergic inputs to DBCs and PV-DTCs by group III mGluRs

The GABAergic inputs of the 2 cell types were differentially affected by group III mGluR activation by LAP4. Spontaneous IPSCs in some PV-DTCs were suppressed as predicted from recordings in rodents, as group III mGluRs inhibit both evoked and spontaneous GABAergic synaptic transmission in vitro (Bonci et al. 1997; Schrader and Tasker 1997; Mitchell and Silver 2000; Wittmann et al. 2001; Matsui and Kita 2003; Turner and Salt 2003; Valenti et al. 2003; Kogo et al. 2004; Panatier et al. 2004; Rusakov et al. 2004; Chu and Moenter 2005; Giustizieri et al. 2005; Cuomo et al. 2009; Summa et al. 2013) and in vivo (Salt and Eaton 1995; Marabese et al. 2005; Naie and Manahan-Vaughan 2005; MacInnes and Duty 2008). Surprisingly, spontaneous GABAergic input to some DBCs was enhanced. This may not be a direct effect on their monosynaptic input synapses, as voltage-gated Na channels were not blocked in our experiments, thus the recorded sIPSCs represented both AP-dependent, and AP-independent GABA release from presynaptic terminals in the network. Furthermore, glutamatergic synaptic transmission was intact, thus group III mGluR action at glutamatergic synapses may have also contributed to the observed effects. Thus, the effects were not restricted to monosynaptic inputs. Facilitation of sEPSCs by group III mGluR activation was reported in layer V neurons in the entorhinal cortex (Evans et al. 2000; Woodhall et al. 2007) and the amygdala (Ren et al. 2011) of rats in vitro, under conditions similar to ours with intact GABAergic neurotransmission. In our experiments, the changes in frequency of sIPSCs were not accompanied by changes in amplitude in either cell type, indicating a presynaptic site of action, consistent with the presynaptic location of group III mGluRs (Shigemoto et al. 1996, 1997; Dalezios et al. 2002; Somogyi et al. 2003). The most likely explanation for the observed facilitation of sIPSCs is a selective disinhibition of a specific GABAergic input to DBCs through network effect. Such a mechanism may also explain the observations of apparent facilitation of neurotransmitter release by group III mGluRs, as measured by microdialysis in other species (Marabese et al. 2005; Li et al. 2008).

The sources of GABAergic innervation of CB+ DBCs are not known. Immunohistochemical studies have shown that CB+ neurons in the monkey visual and temporal cortex receive synaptic input from CR+ neurons and one-third of them receive synaptic inputs from PV-positive neurons (DeFelipe et al. 1999). Because GABAergic inputs to PV-DTCs in humans are suppressed by group III mGluR activation, which may lead to their disinhibition, they could contribute to the increased GABAergic synaptic input to DBCs. The GABAergic inputs to PV-DTCs may include VIP-expressing neurons, a subset of which are known to selectively innervate other GABAergic neurons (Acsády et al. 1996), including those that express PV in the neocortex of rodents (Dávid et al. 2007; Hioki et al. 2013). Consistent with this hypothesis, VIP-expressing neurons in nonhuman species have been shown to express high levels of group III mGluRs, including both the high affinity subtype, mGluR8, as well as the low affinity mGluR7 (Dalezios et al. 2002; Katona et al. 2020). Multiple datasets of single cell RNA sequencing in human neocortex also show selective expression of mGluR8 in some VIP-, SM-, and PV-expressing GABAergic transcriptomic cell types (https://portal.brain-map.org/atlases-and-data/rnaseq).

### Potential roles of DBCs in the neocortex

The physiological activity of DBCs in vivo is not known, but they form a significant radial GABAergic axon system (Somogyi et al. 1981). Any hypothesis for their roles must take into account 2 unique features of CB+ DBCs, namely, the striking radial “horsetail” axon and the high proportion of dendritic spine output synapses. The regularly distributed CB+ or “horsetail” axon bundles found ~15–30 𝜇m apart horizontally (DeFelipe et al. 1990; Peters and Sethares 1997; Ballesteros Yáñez et al. 2005) and spanning layers III–V in primates, including humans, highlights the modular (Szentágothai 1975) and columnar organization in the neocortex (Hubel and Wiesel 1977; Mountcastle 1997). The radial GABAergic CB+ axon bundles appear to run together with bundles of myelinated axons (DeFelipe et al. 1989; Peters and Sethares 1997) adjacent to radially aligned pyramidal cells forming “minicolumns” (Mountcastle 1997) as well as the radial apical dendrite bundles (Peters and Sethares 1996, 1997) through multiple layers. The myelinated axon bundles include, but are not restricted to, the axons of some of the pyramidal cells (Peters and Sethares 1996), and some other pyramidal cells in layers II–III, which have unmyelinated axons making numerous glutamatergic synapses as they descend through the layers. The fine-grained spatial relationships of these components however are not yet clear. In addition, we have shown that multiple individual DBCs, including CB+, CB−, and CR+ cells, with diverse firing patterns and dendritic structure contribute to radial axonal bundles, and further components remain to be explored. All electron microscopic studies agree (Somogyi and Cowey 1981; DeFelipe et al. 1989, 1990; Peters and Sethares 1997) that apical dendritic shafts are not targeted by DBCs, but it cannot be excluded that spines originating from them are innervated. Most dendritic spines and dendritic shafts postsynaptic to DBC GABAergic terminals are probably basal dendrites and/or the oblique dendrites originating from apical dendrites. We hypothesize that the radial beam of high-density GABA release sites from DBCs follows and converges on the same postsynaptic targets as the radial beam of descending pyramidal cell axon terminals. Thus, DBCs are in a position of modulating the effect of the glutamatergic pyramidal cell synapses. This modulation may be inhibitory through both pre- and postsynaptic effects as well as tonic inhibition through extrasynaptic GABA receptors. In turn, the glutamate released by the pyramidal cell terminals may inhibit GABA release through presynaptic mGluRs and assist synaptic plasticity of the glutamatergic synapse (Klar et al. 2015). Several DBC axonal bundles (7–45) would cross the dendritic field of any given radial pyramidal cell column, segmenting the dendritic field and forming a “frame” in which the different bundles may carry information about different horizontal locations in the representational space. If the postsynaptic basal and oblique dendrites of different pyramidal cells in the radial column were aligned at similar angles as viewed from the pia, the DBC axon bundles running through them would shape the responsiveness of the subset of dendrites at similar angles within the column. The preferential innervation of dendritic spines by CB+ DBCs suggests that they regulate glutamatergic synaptic plasticity of some inputs, one of which, as we suggest above, is from the column of pyramidal cells with cell bodies next to the axon bundle. Modeling hypothetical DBC effect on synaptic plasticity suggests that the result would be highly localized to the innervated dendritic column (Bar-Ilan et al. 2013). The CR+ DBCs may modulate signal processing in interneuron dendrites passing through the same radial column. In sensory cortical areas, dendritic spines receiving co-tuned synaptic input are dispersed across the dendritic tree of pyramidal cells, (Jia et al. 2010; Chen et al. 2011), although the clustering of functionally related inputs on neighboring spines at a fine spatial scale can be implemented (Scholl et al. 2017). The “horsetail” axons of DBCs are particularly well suited to modulate synaptic inputs on neighboring spines along restricted dendritic segments of multiple radially aligned pyramidal cells. DBC Innervation on spine heads and/or spine necks might contribute to compartment restricted activation of dendritic spines reported in vivo (Cornejo et al. 2022) and to potential selective pairing between glutamatergic and GABAergic inputs. The enhanced inhibition of DBCs by the action of presynaptic group III mGluRs in the network that we observed could lead to selective disinhibition of innervated dendritic spines on different dendritic segments of a pyramidal cell and provide a mechanism for the functional clustering of neighboring dendritic spines, contributing to their stimulus selectivity (Wilson et al. 2016).

## Author’s contributions

IPL, RF, MF, EH, MH, SH, and PS designed experiments, collected and analyzed data; PP, RS, LL, OA, and GT provided samples, clinical, and management services; all authors edited and approved the manuscript.

## Supplementary Material

supplementary_material_bhac195

## Footnotes

1
http://www3.mpibpc.mpg.de/groups/neher/index.php?page=aboutppt
